# Digital questionnaire response time (DQRT): A ubiquitous and low-cost digital assay of cognitive processing speed

**DOI:** 10.3758/s13428-025-02727-x

**Published:** 2025-06-17

**Authors:** Vanessa Teckentrup, Anna M. Rosická, Kelly R. Donegan, Eoghan Gallagher, Anna K. Hanlon, Claire M. Gillan

**Affiliations:** 1 School of Psychology and Trinity College Institute of Neuroscience, Trinity College Dublin, Dublin 2, Ireland; 2 Global Brain Health Institute, Trinity College Dublin, Dublin 2, Ireland

**Keywords:** Survey response times, Paradata, Cognition, Processing speed, Ecological momentary assessment, Smartphone-based assessment, Ambulatory assessment

## Abstract

Digital survey tools have all but replaced paper and pen in the psychological sciences, and consequently new forms of potentially useful research paradata are now routinely gathered. A particularly common byproduct of research is questionnaire timestamps, which some have suggested can be used as a measure of cognitive function. Here, we conducted a comprehensive validation of this measure, which we call the “digital questionnaire response time,” or “DQRT.” Using data from *N* = 2,977 users of a smartphone app, we first ran a data-driven bootstrapping approach to examine how best to quantify DQRT. DQRT was slower in older adults (*r* = 0.26) and in those with lower educational attainment and socioeconomic status. Testing the association between DQRT and working memory (range *r* = 0.11–0.14), model-based planning (range *r* = 0.03–0.06), and processing speed (range *r* = 0.29–0.39) across cross-sectional and longitudinal subsamples, we found support for a cognitive characterization of DQRT as a measure of cognitive processing speed. DQRT was more strongly correlated with nine out of 13 lifestyle and health factors, and four out of nine mental health factors than a task-based measure of processing speed. DQRT showed good test–retest reliability, and associations between DQRT and task-based processing speed were higher within individuals (*r* = 0.35) than between individuals (*r* = 0.25). Finally, we highlight substantial, but addressable, potential confounds inherent in the measure. We conclude that DQRT has important limitations, but overall can serve as a valid and reliable index of cognitive processing speed that can be gathered at unprecedented scale, unobtrusively, and repeatedly, during a variety of real-world digital behaviors.

**Supplementary Information:**

The online version contains supplementary material available at 10.3758/s13428-025-02727-x.

## Introduction

Cognitive performance is traditionally assessed using validated neuropsychological tests, completed in person in well-controlled laboratory settings. Although this is considered a gold standard methodology, it limits the scale (Button et al., 2013) and diversity (Rad et al., 2018) of the samples that can be studied. For this reason, digital assessment methods have gained popularity (Gillan & Rutledge, 2021), allowing increasingly large groups of people to complete cognitive tests and self-report questionnaires from remote locations (Germine et al., 2012). These digital approaches have proven particularly useful for self-report assessments, allowing researchers to capture within-individual variation across days, weeks, and hours, flexibly and in real-life settings (Hamaker & Wichers, 2017), improving ecological validity (Shiffman et al., 2008) and fueling new forms of clinical research (Lee et al., 2023; Schleider et al., 2022). For cognitive tests, ambulatory assessments are also on the rise (Hawks et al., 2023), but there are challenges—in particular, the elevated burden associated with completing (often lengthy) cognitive tasks (He et al., 2023) caps the quality of data that can be gathered, who we can study, when we can study them, and how often.

Digital paradata hold considerable potential to address this problem. Paradata are a byproduct of digital data that are acquired without active user engagement and include information on the context and process by which the data have been generated (Couper & Kreuter, 2013). One common example of paradata are timestamps of data entry, which have begun to be utilized for research purposes, including to estimate diurnal effects (Bedder et al., 2023) and disrupted circadian rhythms (Lin et al., 2023). These timestamps have also been used to compute reaction times, which can aid in assessing the quality of data from online test-takers (Kreuter, 2013) or serve as an index of the cognitive effort required to answer a survey item (Höhne et al., 2017). Crucially, recent research has suggested that these response times may also serve as a marker of individual differences in cognitive ability itself (Junghaenel et al., 2022).

In perhaps the most comprehensive investigation of this to date, the association between survey response times and cognitive test scores was assessed both cross-sectionally and longitudinally across lags of up to 6.5 years in over 6,000 individuals from an internet panel (Junghaenel et al., 2022). This study found that in terms of cognition, survey response times mapped individual differences in task-switching latencies, and to a lesser extent, verbal and quantitative reasoning. Using the same data but restricting the sample to those aged 50 and above, response times were associated with cognitive impairment and even predicted the likelihood of worse cognitive impairment up to 6.5 years later (Schneider et al., 2023b). Studies using brief ecological momentary assessment (EMA) items have provided further evidence for a link between survey response times and cognitive processing speed and additionally showed that they have good reliability over 2 weeks (Hernandez et al., 2023; Roque et al., 2020). Finally, studies have shown that survey response times are slower in clinical syndromes characterized by cognitive deficits. One example is schizophrenia, where response times predicted both current (Raugh et al., 2021) and future symptom scores (Torous et al., 2018), indicating that survey response times could be capturing cognitive slowing which is observed transdiagnostically (Nigg et al., 2017). Together, these data suggest that survey response times could be a transformative new avenue for studying cognition “in the wild” (Ibanez, 2022) and at an unprecedented scale.

Although there is tremendous potential in what we will refer to here as the “digital questionnaire response time” or “DQRT,” there are several open questions. First, which specific cognitive constructs does DQRT capture: reaction time-based measures of processing or more complex aspects of executive function? It is currently unclear how much data is required to generate a reliable estimate of DQRT, and how it changes with practice and time. Task-based assessments, for example, indicate that lower cognitive performance is linked to higher age (Heiskanen et al., 2024), lower educational attainment (Wilson et al., 2009), and lower socioeconomic status (Schrempft et al., 2023). Similarly, there is evidence indicating that cognitive performance fluctuates throughout the day in line with endogenous biological rhythms (Blatter & Cajochen, 2007) and individual sleep–wake cycles (Schmidt et al., 2007). Furthermore, impaired task-based cognitive performance has been linked to a set of modifiable risk factors for later cognitive decline (Livingston et al., 2020) including lifestyle choices such as smoking (Ernst et al., 2001) or less exercise (Chang et al., 2012), physical health factors such as cardiovascular disorders (Liebel et al., 2017) or diabetes (Moheet et al., 2015; Sadanand et al., 2016), and mental health including psychomotor slowing in depression (McDermott & Ebmeier, 2009). In comparison, associations between cognitive performance and gender have been shown to be heterogeneous and potentially domain-specific (Esposito & Giofre, 2025; Hyde, 2016). For DQRT, despite its potential to be used as a passive proxy of cognitive function (and potentially decline) over time, we know little about its demographic, cognitive, and clinical correlates. Relatedly, no studies to date have examined whether DQRT is confounded by questionnaire content and structure, which pose a threat to its validity in certain contexts. In addition to these open questions, we note that there is no consensus around how DQRT should be calculated, creating space for researcher degrees of freedom. The present study aimed to fill this gap and develop a robust pipeline for the derivation of DQRT and report on its psychometric properties. In addition, we aimed to explore cognitive and individual difference correlates to provide a foundation for future clinically oriented research. To do this, we leveraged a large existing dataset comprising *N* = 2,977 individuals who completed questionnaires and three cognitive tasks measuring cognitive processing speed, working memory, and model-based planning on a research smartphone app (Neureka) cross-sectionally, and *N* = 84 individuals who did so longitudinally.

## Methods

### Participants

We included citizen scientists who had downloaded the smartphone research app Neureka between June 2020 and November 2023. To be included for analysis, participants had to be at least 18 years of age and indicate English as their first language (see Fig. S1 for a flowchart). All study-related inputs were done via touchscreen, and participants used their personal handheld mobile devices, so screen size was not normed for this study. All participants gave informed consent for their data to be included in analyses. A total of *N* = 3,278 participants had completed the Risk Factors science challenge. Of these, *N* = 2,977 (*M*_age_ = 46.6±14.5, 66% women) indicated English as their first language (Survey Set 1). A subset of *N* = 991 (*M*_age_ = 46.0±14.5, 68% women) additionally completed the My Mental Health science challenge (Survey Set 2). A total of *N* = 460 participants had completed the baseline and the first assessment day of the Brain Changer science challenge. Of these, *N* = 423 (*M*_age_ = 49.9±13.0, 71% women) indicated English as their first language (EMA Set). A subset of *N* = 84 (*M*_age_ = 55.9±10.8, 70% women) additionally completed a minimum of 15 days of repeated assessments (out of possible 28) comprising a gamified trail making test (once every second day) and ecological momentary assessment (twice per day). The Neureka project was approved by the research ethics committee of the School of Psychology, Trinity College Dublin (approval number SPREC072019-01). Data collected through the Neureka app are stored and processed in line with the EU General Data Protection Regulations.

### Experimental procedure

Participants were recruited online, downloaded the smartphone research app Neureka, and completed the sign-up process which included questions pertaining to their demographics, assessing their age, gender, education level, and first language. After sign-up, users are free to explore Neureka, which offers a set of science challenges that consist of modules containing surveys, quizzes, and gamified experimental tasks focusing on different aspects of brain health. This study was not preregistered.

### Science challenges

#### Risk Factors

The Risk Factors challenge aims to measure lifestyle and demographic risk factors for dementia (Livingston et al., 2020). Participants complete three gamified cognitive tasks and 12 questionnaires organized into six modules which are presented in random order with the constraint that a task will always be delivered first, followed by three questionnaire modules, followed by the second task, followed by another three questionnaire modules, and ending with the third task.

#### Questionnaires: Lifestyle, demographics, and health (Survey Set 1)

The Risk Factors challenge contains 12 questionnaires. Of these, we selected seven for analysis, comprising 68 items (Survey Set 1) probing the following: depression (Center for Epidemiologic Studies Depression Scale, CES-D, Radloff, 1977), 20 items; loneliness (UCLA Loneliness Scale, Russell et al., 1978), 20 items; social network (Lubben Social Network Scale, Lubben et al., 2006), six items; hearing handicap (Hearing Handicap Inventory–Screening Version, HHIE-S, Weinstein & Ventry, 1983), 10 items; cognitive fluctuations (Mayo Cognitive Fluctuations Questionnaire, Ferman et al., 2004), four items, attitude toward own aging (Attitude Toward Own Aging, ATOA, Lawton, 1975), five items; and exercise (Godin Leisure-Time Exercise Questionnaire, Godin, 2011), three items. The remaining questionnaires were excluded because they comprised single items, were not standardized scales, or had conditional structures, i.e., where some questions were dependent on prior answers. These measured hearing loss, family history of dementia, smoking, subjective socioeconomic status (Adler et al., 2000), and custom items assessing lifestyle dementia risk factors (Livingston et al., 2020).

#### Star Racer (Trail Making Test): Trails A and B

**Fig. 1:**
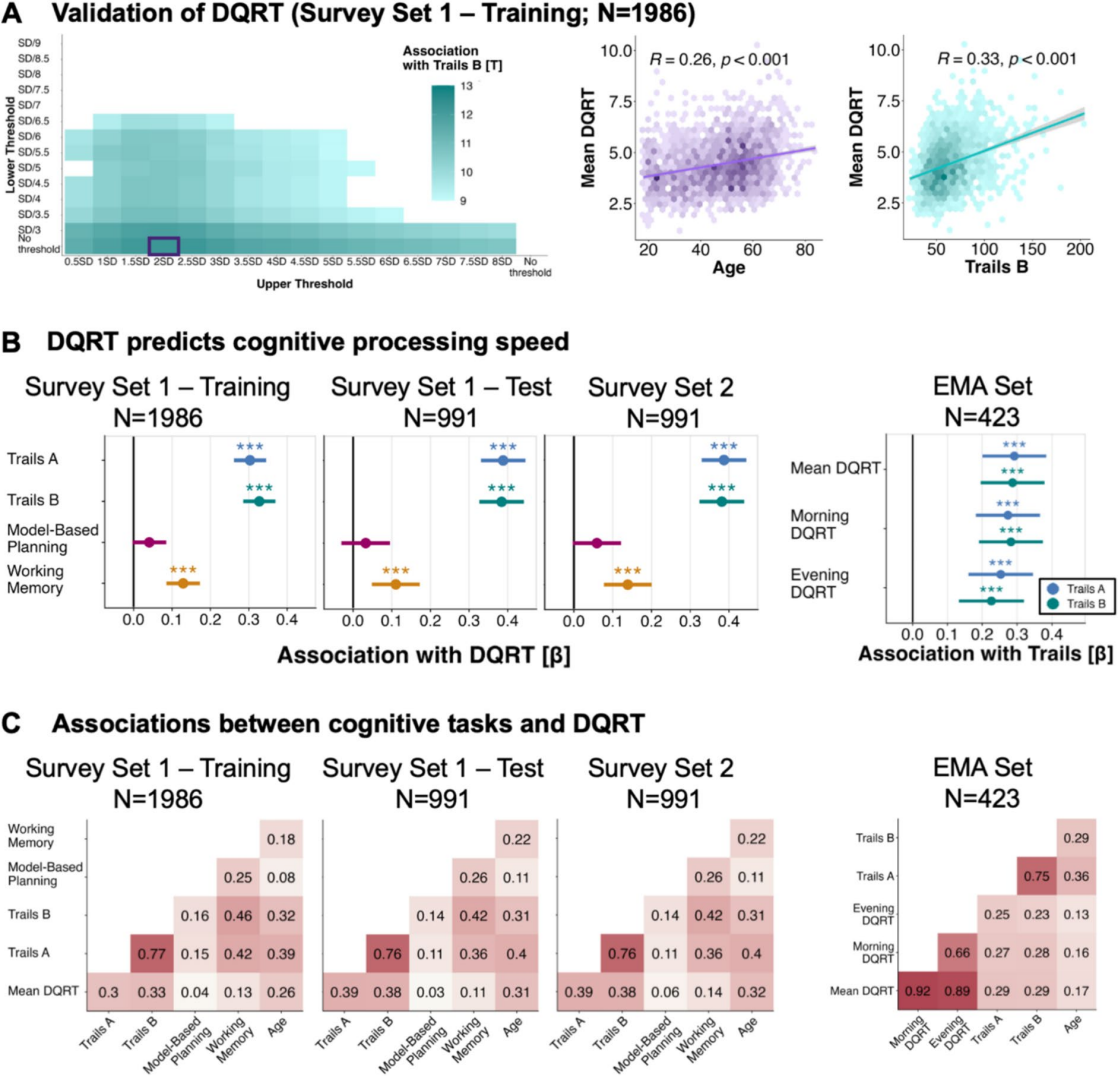
Associations between digital questionnaire response time (DQRT) and gamified tasks measuring cognition.** A** Threshold values for DQRT calculation were determined in a training sample (Survey Set 1–Training, *N* = 1,986) using a data-driven bootstrapping approach that maximized the association with trails B (Trail Making Test). The heatmap shows *t* values for the association between DQRT and trails B depending on combinations of upper (*x*-axis) and lower (*y*-axis) threshold values. The purple rectangle marks the combination of threshold values with the highest *t* value. Applying these threshold values to the Survey Set 1 training sample, DQRT was correlated with trails B at *r* =.33 (right column) and age at *r* =.26 (left column). **B** DQRT predicted trails A, trails B, and working memory, but not model-based planning, in the training set (Survey Set 1–Training), in the held-out test set (Survey Set 1–Test), and when calculated from a separate set of questionnaires (Survey Set 2). DQRT calculated from brief ecological momentary assessment (EMA) items was associated with trails A and trails B (EMA Set), irrespective of whether they were measured in the morning or the evening, or averaged across both timepoints. Point estimates are standardized beta coefficients, and error bars depict 95% confidence intervals. **C** Correlation plots depicting inter-correlation of all cognitive measures with age, for all datasets. * *p* <.05; ** *p* <.01; *** *p* <.001

Star Racer is a gamified version of the Trail Making Test (TMT, Bowie & Harvey, 2006; Spreen & Strauss, 1998), which is commonly used to measure processing speed and cognitive flexibility as a facet of neuropsychological impairment in clinical settings. A detailed description and validation of Star Racer has previously been published (Rosická et al., 2023). Briefly, 25 blue-colored stars are placed on the screen, and participants are asked to tap on them in ascending order as fast as they can (Fig. S2). Like the TMT, Star Racer has two versions. In trails A, each star has a number from 1 to 25, and participants are required to tap on the stars in ascending numerical order. In trails B, the stars show both numbers (between 1 and 13) and letters (between “a” and “l”). Participants are then asked to tap on the stars in ascending numerical and alphabetical order, alternating between numbers and letters. Version A of the task has commonly been linked to processing speed, whereas version B has been proposed to reflect cognitive flexibility (Bowie & Harvey, 2006). However, the two versions are highly correlated (*r* =.74; Tombaugh, 2004), indicating an overlap in the cognitive processes they capture (Varjacic et al., 2018). Participants collect points for each correctly selected star and lose points for choosing an incorrect star. They receive feedback in both cases: correctly selected stars turn pink, whereas incorrectly selected stars turn light purple and shake. Errors are not scored for analysis purposes; as per the standard paper-and-pencil version, errors result in increased time to complete a run. Participants were asked to first complete two practice rounds with eight stars per screen (once for version A, once for version B). After this, they completed two rounds in which the stars were hard-coded to be in positions similar to the paper-based TMT. The remaining four runs (twice for version A, twice for version B) used randomly generated star locations. For analysis, the outcome of interest was the average of the time needed to complete each of the three runs of trails B (25 stars per screen). Given that completion times for trails A and B are highly correlated, we focused on trails B runs for most of the analyses, because trails A does not feature in our EMA dataset. Nonetheless, results for trails A runs are presented in Fig. 1 and are highly similar to results reported for trails B. As participants completed Star Racer remotely, we applied cutoffs to exclude runs that were indicative of inattentive players. For trails B, we excluded runs exceeding 300 s, and for trails A, we excluded runs exceeding 100 s. These cutoffs are conservative compared to values acquired for the oldest and least educated group in a normative study (Tombaugh, 2004), as they reflect double the median completion time for trails A (*Mdn* = 54.5 s) and trails B (*Mdn* = 142.5 s) in that group.

#### Cannon Blast (two-step task): Model-based planning

Cannon Blast is a gamified version of the two-step reinforcement learning task (Daw et al., 2011) which estimates a participant’s “model-based” tendency, representing the extent to which they make decisions using a mental map of action–outcome associations. Briefly, participants shoot balls to hit and collect as many diamonds as possible. Balls are drawn from one of two containers, with the left container having more purple balls (80%) and the right container having more pink balls (80%). Balls drawn from a container either are good balls, which can be used to collect the diamond (reward trial), or disintegrate upon firing, making it impossible to collect the diamond (unrewarded trial). The outcome of interest is the model-based index, which is calculated using a hierarchical logistic regression model, predicting the binary choice between the two containers as a function of reward (good or bad ball) and transition (which color was shot from the chosen container). A detailed description and validation of Cannon Blast has been published previously (Donegan et al., 2023), and further information is available in the methods section of the online supplement (Fig. S3).

#### Memory Match (visual short-term memory binding task): Working memory

Visual working memory performance was assessed using Memory Match, a game loosely based on a previously published visual short-term memory binding task (Parra et al., 2010). The outcome of interest for Memory Match is an individual’s mean accuracy, calculated as the proportion of correct responses given the total number of responses across 24 trials that vary in difficulty. A detailed description and validation of Memory Match has been published previously (Rosická et al., 2023), and further information is available in the methods section of the online supplement (Fig. S4).

#### My Mental Health (Survey Set 2)

The My Mental Health science challenge in Neureka is a set of nine questionnaires with 209 items in total which participants complete at their own pace. Questionnaires are presented in randomized order. The questionnaires tap into transdiagnostic aspects of mental health (Gillan et al., 2016; Wise et al., 2023) and measure compulsivity (Obsessive Compulsive Inventory–Revised [OCI-R], Foa et al., 1998), depression (Self-Rating Depression Scale [SDS], Zung, 1965), apathy (Apathy Evaluation Scale [AES], Marin et al., 1991), social anxiety (Liebowitz Social Anxiety Scale [LSAS], Liebowitz, 1987), eating disorders (Eating Attitudes Test [EAT-26], Garner et al., 2009), alcohol use (Alcohol Use Disorder Identification Test [AUDIT], Saunders et al., 1993), trait anxiety (State-Trait Anxiety Inventory–Trait [STAI-T], Spielberger, 1983), impulsivity (Barratt Impulsiveness Scale [BIS], Patton et al., 1995), and schizotypy (short scales for measuring schizotypy [SSMS], Mason et al., 2005).

#### Brain Changer (science challenge completed by individuals in the EMA Set)

The Brain Changer science challenge follows participants for 56 days and requires them to complete ecological momentary assessments twice a day in the morning and the evening. Every second day, participants additionally complete two cognitive tasks, a perceptual decision-making task (Fox et al., 2024; not analyzed in this work) assessing metacognitive performance, and Star Racer–trails B, which has been proposed to reflect cognitive flexibility but, in line with its high correlation with trails A, also captures processing speed (Varjacic et al., 2018). Every second week, participants additionally completed the Quick Inventory of Depressive Symptomatology–Self-Report (QIDS-SR-16, Rush et al., 2003). To start the challenge, participants completed a baseline assessment that relates to lifestyle factors, depression symptoms, mood, anxiety, stress, transdiagnostic factors, self-report measures related to mental health, and the two cognitive tasks. EMA items were adapted from the QIDS-SR-16 and in the morning included 17 items measuring sleep quality and duration, appetite, perceived worthlessness, sadness, ability to concentrate, decision-making, perceived slowness, tiredness, loss of interest, anhedonia, restlessness, perceived guilt, worry, irritation, anxiety, and perceived stress. Assessments in the evening used a reduced set of 14 items, as sleep quality, sleep duration, and appetite were assessed only once a day. All items except for the appetite item were rated using a slider on the same 11-point visual analogue scale ranging from 0 (*not true at all*) to 10 (*extremely true*). Appetite was rated using a slider on an 11-point visual analogue scale ranging from 0 (*low*) to 10 (*high*). EMA items were presented in randomized order, but morning-only items were always presented first. For the analyses reported in this work, only the baseline and bidaily assessments of Star Racer–trails B and each available EMA assessment for each individual in the EMA Set were included.

### Data analysis

All data were preprocessed and analyzed using R statistical software (v4.3.2, R Core Team, 2021), and all scripts used to generate the results and figures can be accessed on the Open Science Framework: 10.17605/OSF.IO/HNCSG.

#### Digital questionnaire response time

For each of the questionnaires included in the analysis, we extracted the item-level timestamps, which had a lower bound sensitivity of 1 s. If participants had completed one of the science challenges that was not a longitudinal EMA assessment (i.e., Survey Set 1 and Survey Set 2) in the app multiple times, we used their first set of responses. DQRTs were calculated across sets of questionnaires within a science challenge as well as for individual questionnaires, depending on the analysis. Careless responding or interruptions could lead to response times that were artificially short or long. To address this in a principled way, we adopted a data-driven approach to determine lower and upper thresholds expressed in units of the median ± a certain number of standard deviations (*SD*). We chose standard deviation to unbind this analysis from the particulars of our questionnaires, and therefore facilitate comparison with other datasets in the future. First, we split the data from Survey Set 1 into training (*N* = 1,986) and test (*N* = 991) sets. As the standard deviation is strongly affected by outliers, we first calculated DQRTs for the full training set, applying a lenient initial threshold, removing all single-item RTs > 900 s. We then used a bootstrap approach to prevent overfitting of the threshold values to the training set. Drawing from the training sample with replacement, we generated 1,000 bootstrapped samples of the same size. Within each of the 1,000 bootstrapped samples, we calculated the median DQRT. Then we ran each bootstrapped sample through every combination of a prespecified set of lower and upper threshold values. The upper threshold was defined as $median DQRT+(modifier \times SD\left(DQRT\right))$. The set of modifiers we tested ran from 0.5 to 8 in increments of 0.5. We also included the option to apply no upper threshold at all. The lower threshold was given by $median DQRT-\left(SD\left(DQRT\right)/modifier\right)$. As negative DQRTs are not defined, the use of fractions of *SD* for the lower threshold ensured that the threshold value would not fall below zero. The set of modifiers we tested ran from 3 to 9 in increments of 0.5. Again, we included the option to apply no lower threshold at all. For each combination of lower and upper thresholds, we removed item-level RTs which fell outside of the thresholds. Then we calculated the DQRT by averaging the single-item RTs across all questionnaires and ran linear models predicting DQRT by cognition as indexed by trails B (Star Racer). We then extracted the *t* value from each model and averaged the *t* value within each combination of lower and upper thresholds across all 1,000 bootstrap runs. Finally, we determined the upper and lower threshold values that were associated with the highest average *t* value. For the lower threshold, the highest *t* value was achieved when applying no threshold. For the upper threshold, the highest *t* value was achieved when applying a threshold of $median DQRT+(2 \times SD\left(DQRT\right))$. To generate the final set of DQRTs that was subjected to further analyses, we removed item-level DQRTs which fell outside of these threshold values. The resulting distributions of DQRT for each questionnaire are presented in Fig. S6.

#### Validity of DQRT as a measure of cognition

To test the relationship between DQRT and cognitive task outcomes, we first ran correlation analyses including trails A, trails B, model-based planning, and working memory separately for the training set based on Survey Set 1 (*N* = 1,986). Next, we replicated these findings in holdout data not involved in the optimization process above (*N* = 991). Finally, we tested for generalizability by running the same analysis for DQRTs estimated from two other sections of the app. The first was a section comprising questions about mental health (Survey Set 2), from which DQRTs were extracted and related to the same task measures as the training and test sets. The second was another section of the app where DQRTs were estimated from EMA items (EMA Set) and cognition was assessed entirely independently of the original validation (*N* = 423). As measures of cognition show a correlation with age, we ran a linear model predicting DQRT from age in all data partitions. Finally, we tested whether the associations between DQRT and cognition were dependent on the content of the questionnaires DQRT originated from. We calculated mean DQRT for each questionnaire separately for both Survey Set 1 and Survey Set 2. We then used the questionnaire-specific mean DQRTs as predictors in linear models predicting trails A and trails B game times, model-based planning, and working memory.

### Stability of DQRT

To serve as a time-efficient proxy for task-based assessments of cognition, the number of items required to achieve a stable association between cognitive outcomes and DQRT needs to be low. Hence, we tested how many questionnaire items were necessary to achieve this stable association in both Survey Set 1 and Survey Set 2. For this, we calculated mean DQRT based on two items (minimum number of items necessary) and reran the linear models predicting trails B game time, model-based planning, and working memory from mean DQRT. For each subsequent iteration, we added one more DQRT to calculate the mean DQRT and reran the models again until all DQRTs were included. To obtain more comparable results for the two samples, we restricted this iterative process to 61 steps (number of steps in Survey Set 1) in Survey Set 2. To prevent order effects, we repeated this process 100 times, each time shuffling the order of the questionnaires that the items were drawn from. We then averaged the beta estimates and standard errors for each cognitive outcome across the 100 shuffled runs. To identify the number of items needed for a stable association between cognition and DQRT, we assumed that the predictability of trails B time by DQRT would follow a logarithmic growth curve characterized by a positive rate of change which asymptotes to zero once adding more items no longer increases predictability (Buyalskaya et al., 2023). Hence, we fitted an asymptotic predictability curve with $E\left(s\right)= a-b{e}^{-cs}$, where $E$ is the beta estimate for trails B, $s$ is the respective step in the iterative process of adding items, $a$ is the asymptotic level of the trails B estimate, $a-b$ is the starting value, and $c$ is the rate of change, with higher values indicating faster change. We then calculated the number of iterative steps it took for $E\left(s\right)$ to reach 95% of its estimated asymptote $a$. As uncertainty around the number of items needed to achieve a stable association between DQRT and trails B is linked to sample size, we ran an additional validation analysis. We used bootstrap sampling to generate new samples from Survey Set 2 which allowed us to explore higher ranges of potential item numbers due to the overall higher number of items included compared to Survey Set 1. The size of newly generated samples ranged from *N* = 100 to the full *N* = 991 in steps of 100. Again, we fitted asymptotic predictability curves 100 times per sample size and calculated the number of iterative steps it took to reach the 95% asymptote, randomizing the order in which items were added in each iteration. We then calculated the mean and standard deviation for the number of iterative steps across the 100 iterations per sample size. Whereas the mean number of iterative steps was expected to vary only slightly across sample sizes, the standard deviation, and hence the upper bound of items necessary for a stable association between DQRT and trails B, was expected to decrease from smaller to larger sample sizes.

#### Test–retest reliability of DQRT

To assess the test–retest reliability of DQRT, we used naturally occurring lags between participants’ completion of different sections of the app. We split the sample into three groups depending on the time lag between the completion of Survey Set 1 and Survey Set 2. These were participants who (1) completed both science challenges on the same day (*N* = 630, 67.1% completed Survey Set 1 first), (2) completed the second science challenge up to 7 days after the first (*N* = 234, 80.3% completed Survey Set 1 first), and (3) completed the second science challenge more than 7 days after the first (*N* = 127, 75.6% completed Survey Set 1 first). We then ran two analyses to calculate within-person reliability: a correlation analysis to assess the linear association and an intraclass correlation (ICC) analysis using a two-way mixed-effects model (single raters, absolute agreement; McGraw & Wong, 1996) to assess agreement between DQRT estimated from the two sections. To assess within-person reliability across several repeated assessments, we then examined data from a subsample who had a minimum of 15 bidaily EMAs (*N* = 84) from the Brain Changer challenge (EMA Set). We again calculated the intraclass correlation coefficient using a two-way mixed-effects model (single raters, absolute agreement). As the EMA Set included repeated assessments of Star Racer, we also provide the ICC for trails B for comparison. Finally, we assessed the within-person versus between-person variability in the association between DQRT and trails B. Previous research has shown that inferences drawn on the group level often do not generalize to the individual level, where effects tend to be weaker (Fisher et al., 2018). Hence, we computed the correlation between DQRT calculated from the EMA Set and Star Racer assessments across assessment days (within subjects) and compared them to correlations calculated across participants (between subjects). As the mean number of repeated assessments was 24, we randomly sampled *N* = 24 participants from the pool of *N* = 84 in the EMA Set to equivalently power the within-individual assessments comparably to assessments between individuals. To prevent bias from sampling, we repeated this process 100 times and took the mean across the 100 runs. Using the EMA Set, we further assessed whether repeated assessments led to a practice effect on mean DQRT or mean trails B completion time. For practice effects we assumed a linear decrease across assessments, meaning that participants would increase their speed with increasing experience. We ran hierarchical linear models predicting mean DQRT or mean trails B completion time from assessment day. To account for repeated assessments within participants, we modeled participant ID as a random intercept. To test for practice effects on the association between mean DQRT and mean trails B completion time, we ran a hierarchical linear model predicting mean trails B completion time from the interaction between mean DQRT and assessment day. Again, we accounted for repeated assessments within participants by modeling participant ID as a random intercept.

#### Association between DQRT and demographics, device type, and time of day

To assess whether DQRT is related to demographic variables beyond age, we ran separate linear models predicting DQRT from gender, educational attainment, socioeconomic status, and device type (iOS/Android) in Survey Set 1 and Survey Set 2. All models included age as a covariate of no interest. Models based on educational attainment, socioeconomic status, and device type additionally included gender as a covariate of no interest. To compare the results to task-based processing speed, we then ran the same models predicting trails B (derived from Star Racer in Survey Set 1) instead of DQRT. Lastly, we tested whether response times differed between assessments completed in the morning versus the evening using the EMA Set. Here, we used a linear mixed model predicting DQRT from time of assessment (morning/evening) and included age, gender, and assessment day as covariates of no interest.

#### Association between DQRT and lifestyle and health factors

We tested whether DQRT relates to individual differences in lifestyle, physical, and mental health factors. We ran linear models predicting the continuous total scores of scales in Survey Set 1 (exercise, cognitive fluctuations, attitude toward aging, hearing handicap, depression, loneliness, social network) and in Survey Set 2 (social anxiety, apathy, schizotypy, trait anxiety, depression, obsessive–compulsive disorder (OCD), impulsivity, eating disorder, alcohol use) from DQRT. We estimated these effects twice, once for DQRT estimated from the same questionnaires from which the total scores were derived, and once for DQRTs estimated from a different set of questionnaires (which may not have been completed on the same day). We ran the same models using trails B game time as the independent variable instead of DQRT to benchmark these results. For binary outcomes in Survey Set 1 (subjective memory problems, smoking, tinnitus, diabetes, hypertension, family history of dementia), we ran similar models using logistic regression instead. To account for differences in exposure to the lifestyle and (mental) health factors, we included both age and gender as covariates in all models.

#### Potentially confounding effects of questionnaire content

We tested whether relationships between DQRT and questionnaire total scores depend on the content of the questionnaire used to estimate DQRT. We calculated DQRT from each questionnaire individually and compared the magnitude of the association between DQRT and total scores to the expected magnitude for the same number of items, but independent content. For this, we bootstrapped the association between questionnaire total scores and 100 randomly drawn DQRT estimates within Survey Set 1 and Survey Set 2, respectively, thereby obtaining a distribution of the expected effect.

## Results

### Estimation and validation of DQRT as a measure of cognitive processing speed

DQRT is calculated as the average time taken to complete a set of individual survey items, across one or more questionnaires. We defined a training set of *N* = 1,986 individuals who completed cognitive tests on the same occasion as they completed surveys pertaining to potential risk factors for dementia (Rosická et al., 2023) (Survey Set 1; 68 items). In a first step, we examined the association between a minimally processed version of DQRT (excluding only responses > 900 s) and three cognitive tests assessing trails A and trails B game times, working memory, and model-based planning. Trails A and B game times derived from a gamified version of the Trail Making Test had the strongest association with DQRT (see Table S1). Due to the high correlation between trails A and B, only one of the measures—trails B—was carried forward and used in a second step to determine optimal cutoffs for trial-wise exclusions using a data-driven bootstrapping approach aimed at maximizing this association (Fig 1A, left). The optimal solution removed responses two standard deviations (*SD*) above the sample median and applied no lower threshold. The mean DQRT in the sample was 4.4 s, *SD* = 1.2 (see Fig. S6 for full distributions over all surveys). Applying these threshold values to the training data, DQRT was significantly associated with trails A (β [CI] = 0.30 [0.26 0.35], *p* <.001), trails B (β [CI] = 0.33 [0.29 0.37], *p* <.001), and to a lesser extent, working memory (β [CI] = 0.13 [0.09 0.17], *p* <.001), but not model-based planning (β [CI] = 0.04 [−0.003 0.09], *p* >.05) (Fig. 1B, C: Survey Set 1–Training). As expected, DQRT also increased with age (β [CI] = 0.26 [0.22 0.30], *p* <.001) (Fig 1A, middle). Applying the threshold values from the training set to a fully held-out test set (*N* = 991), we replicated these results (Fig. 1B, C: Survey Set 1–Test; Table S2). These effects further generalized to DQRT estimated from *N* = 209 items assessing mental health symptoms that were also completed by the held-out sample in a different section of the app (Fig. 1B, C: Survey Set 2; Table S2). Finally, results from these full-form questionnaires generalized to DQRT calculated from much shorter ecological momentary assessment (EMA) items in a separate sample of individuals that had completed the EMA section of the app (*N* = 423). Calculating DQRT from participants’ first day of EMA and relating it to task performance from the prior day, we found the same pattern of results (Fig. 1B, C EMA Set; Table S2), albeit with slightly attenuated effect sizes compared to the other datasets with long-form surveys.

#### DQRT stabilizes quickly

**Fig. 2:**
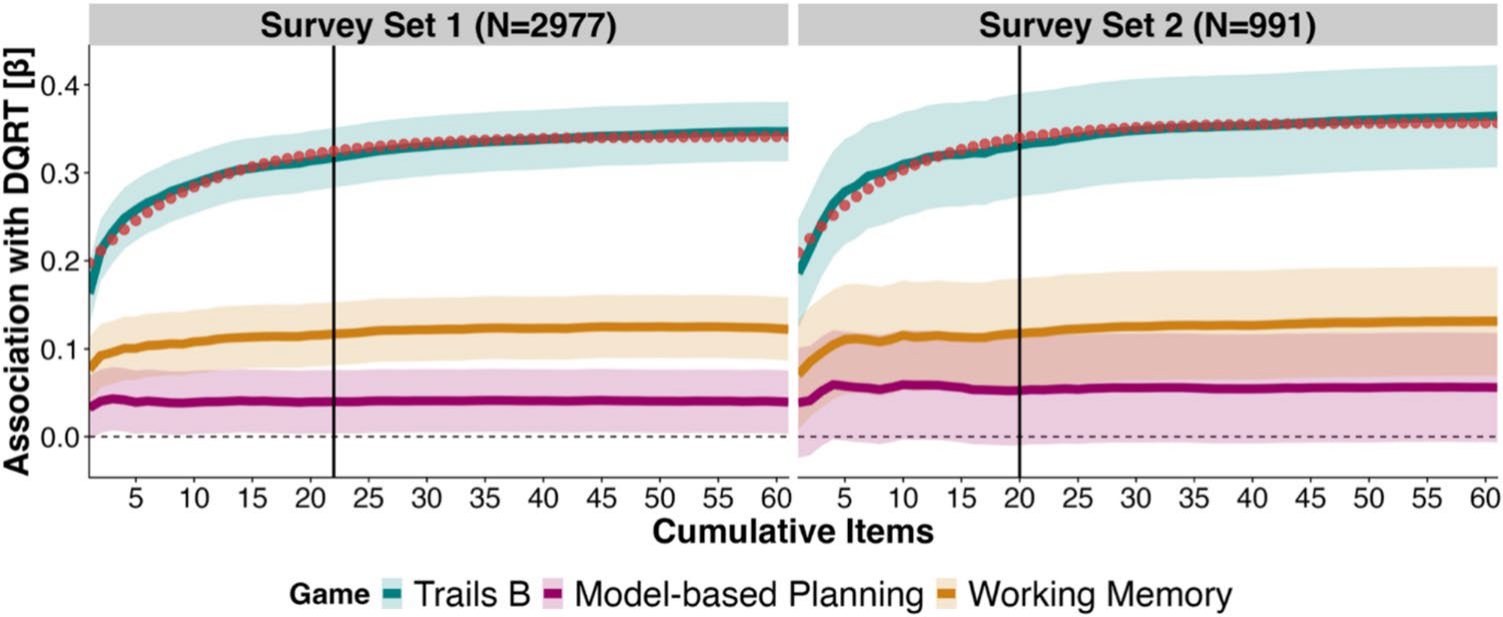
Association between digital questionnaire response time (DQRT) and cognitive processing speed stabilizes quickly. The curves describe the predictive performance of mean DQRT for trails B (teal), model-based planning (pink), and working memory (yellow) across steps of cumulatively adding questionnaire items and recalculating mean DQRT in Survey Set 1 (left) and Survey Set 2 (right), respectively. Fitting a logarithmic function to the curves describing the association between DQRT and trails B (red, dotted line) allows us to identify the point at which the predictability curve reaches 95% of the estimated asymptote, i.e., the number of items necessary to achieve a stable association (black vertical lines). DQRT reaches stability with 22 items in both samples. Colored lines are standardized beta coefficients, and shaded areas depict 95% confidence intervals

To establish how many questionnaire items are necessary to achieve a stable association with trails B game time, we started from two questionnaire items and subsequently added single-item RTs to recalculate mean DQRT until all items were added (*N*_items_ = 68, the maximum number of items in Survey Set 1). As participants received questionnaires in random order and they differed in length, we repeated this process 100 times, each time shuffling the order of questionnaires. We then fitted a logarithmic function to calculate the number of iterative steps it took for the curve reflecting the association between trails B game time and DQRT to reach 95% of its estimated asymptote. The logarithmic function showed an excellent fit to the empirical curves (Survey Set 1, *N* = 2,977: *R*^2^ = 0.96; Survey Set 2, *N* = 991: *R*^2^ = 0.96). The fitted asymptotic value was $a$ = 0.34 and $a$ = 0.36, respectively. For Survey Set 1, 22 items were required, and for Survey Set 2, 20 items were required to reach the point of stabilization (Fig. 2). As uncertainty around the number of items is linked to sample size, we ran an additional validation by bootstrapping subsamples from Survey Set 2 with a sample size ranging from *N* = 100 to the full *N* = 991 in steps of 100, and rerunning the estimation of the asymptotic value 100 times per sample size. We show the mean estimated number of items and standard deviation across sample sizes in the online supplement (Fig. S7). We downsampled data from Survey Set 2 (from 209 to 68 items) for comparability with data from Survey Set 1 but recognizing that fitting the full set of 209 items slightly changes the asymptotic value. When using all 209 items compared to just 22 to calculate DQRT, the estimated asymptotic value rose to $a$ = 0.38 and the association with trails B showed a small but significant increase (β [CI] = 0.05 [0.04 0.05], *p* <.001), amounting to 13.1% change in signal.

#### DQRT is reliable

**Fig. 3:**
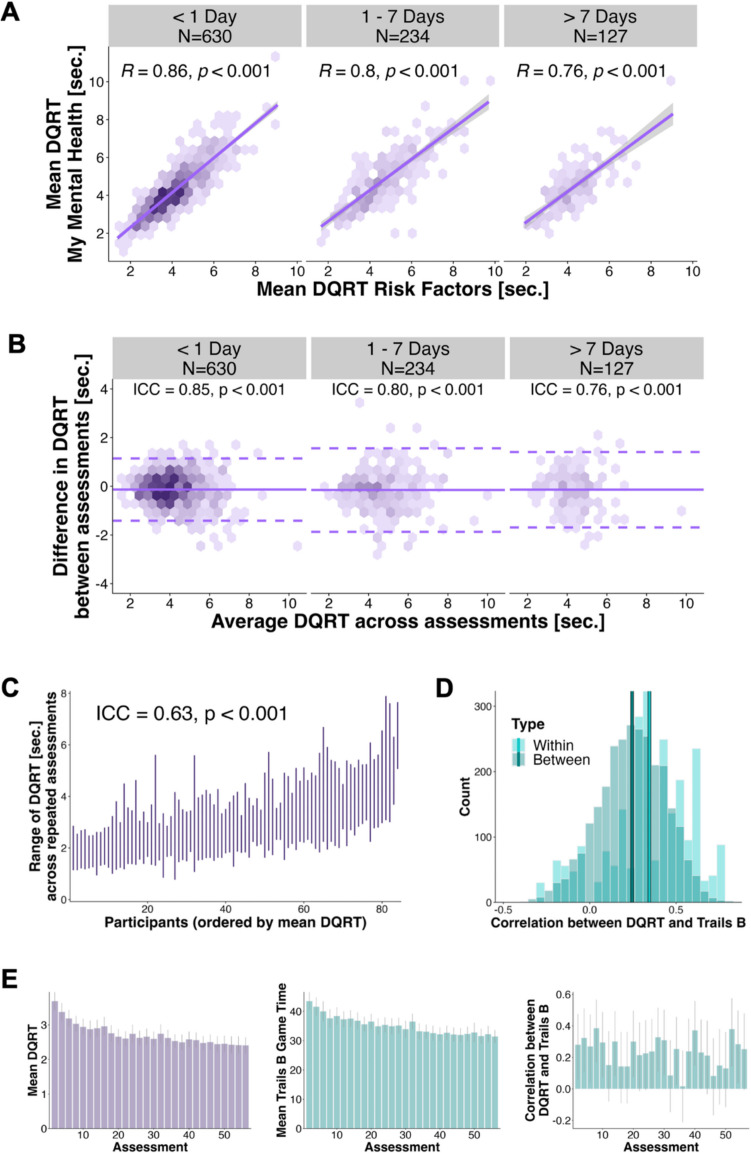
Digital questionnaire response time (DQRT) is reliable.** A** We first assessed within-person reliability using the linear association between mean DQRT from Survey Set 1 and mean DQRT from Survey Set 2, split by time lag. Correlation was good across all splits but decreased with increasing time lag. **B** As different questionnaire sets could result in different (but linearly scaled) values for DQRT, and linear associations do not allow one to assess the degree of concordance between repeated assessments, we calculated the intraclass correlation to measure agreement between mean DQRT from Survey Set 1 and mean DQRT from Survey Set 2, split by time lag. Again, agreement was good across all splits and decreased with increasing time lag. The Bland–Altman plot shows the mean difference in DQRT between measurements (solid purple line) and the associated confidence interval (dashed purple lines). Mean differences were close to zero, indicating low bias, i.e., DQRTs measured via the first questionnaire set were very similar to DQRTs measured via the second questionnaire set. **C** DQRT showed moderate within-person (*N* = 84) agreement across a minimum of 15 repeated assessments and **D** showed similar magnitude and variability for the association with trails B (*N* = 84) when assessed within individuals (across assessments) compared to between individuals (across participants). **E** Both DQRT (left) and trails B game time (middle) decreased over a minimum of 15 repeated within-person (*N* = 84) assessments, indicating practice effects. Similarly, the association between DQRT and trails B showed a small linear decrease over time based on a model predicting trails B game time from an interaction between DQRT and time. Error bars show the 95% confidence interval

To estimate the test–retest reliability of DQRT, we ran intraclass correlation analysis which assessed within-person reliability by comparing DQRTs calculated from Survey Set 1 with DQRT calculated from Survey Set 2, with varying lags between assessments. We split the sample into three groups as a function of number of days between assessments (Group 1: < 1 day; Group 2: 1 day ≥ lag ≤ 7 days; Group 3: > 7 days). For the same day group (*N* = 630, *Mdn*_lag_ = 0.04 days, *range*_lag_ = 0.004–0.99 days), we found good reliability of ICC_(A,1)_ [CI] = 0.85 [0.83 0.88] (*p < *.001). For those with an interval ranging from 1 day to 1 week (*N* = 234, *Mdn*_lag_ = 2.4 days, *range*_lag_ = 1–6.9 days), reliability remained good, ICC_(A,1)_ [CI] = 0.80 [0.75 0.84] (*p* <.001), and this decreased to moderate to good ICC_(A,1)_ [CI] = 0.76 [0.67 0.82] (*p* <.001) for the third group who had a lag of > 1 week between assessments (*N* = 127, *Mdn*_lag_ = 15.9 days, *range*_lag_ = 7.1–547 days) (Fig. 3B).

We further assessed the within-person variability of DQRT by calculating the ICC of DQRT estimated from EMA data gathered every second day for 8 weeks in participants with a minimum of 20 assessments who also had trails B task data on those days (*N* = 84, *M*_NAssessments_ = 24, *range*_NAssessments_ = 15–28). For DQRT, we found a moderate ICC_(A,1)_ [CI] = 0.63 [0.55 0.71] (*p* <.001; Fig. 3B). In comparison, trails B game time was somewhat less variable, with ICC_(A,1)_ [CI] = 0.73 [0.66 0.80] (*p* <.001). As practice effects due to repeated assessments could affect both DQRT and trails B, we assessed whether the assessment day (time) predicted mean DQRT or mean trails B game time, or whether time modulated the association between DQRT and trails B (time × DQRT interaction; Fig. 3D). Both DQRT (β [CI] = −0.25 [−0.28 −0.23], *p* <.001) and trails B game time (β [CI] = −0.24 [−0.26 −0.21], *p* <.001) decreased across repeated assessments. Similarly, the association between DQRT and trails B game time decreased over time, but the effect size was small (β [CI] = −0.03 [−0.05 −0.01], *p* =.003). Finally, we compared the magnitude of within- versus between-person effects on the strength of the association between DQRT and trails B game time (Fig. 3C). To do this, we computed the between-person correlation between DQRT and trails B for each day of the assessment, downsampling the number of individuals to the mean number of days completed for comparability (*M*_N(assessment days)_ = 24 days, *N* = 24 individuals), and repeating this process 100 times with randomly drawn samples of participants. We compared this to each person’s within-person correlation of DQRT with trails B over the same time series. The association between DQRT and trails B was somewhat stronger within individuals (mean *r* = 0.35) than between individuals (mean *r* = 0.25), with similar variability within individuals (*SD* = 0.24) and between individuals (*SD* = 0.19).

#### DQRT is associated with demographics, time of day, and device type

Having already established that DQRT is slower with higher age, we tested whether it relates to other demographic variables. Compared to individuals identifying as cisgender male, individuals identifying as cisgender female (β [CI] = −0.09 [−0.12 −0.05], *p* <.001) and individuals identifying as non-cisgender (β [CI] = −0.13 [−0.26 −0.01], *p* =.03) had faster DQRTs in Survey Set 1 (*N* = 2,977). DQRT was significantly slower with lower educational attainment (β [CI] = 0.17 [0.13 0.20], *p* <.001) and lower socioeconomic status (β [CI] = 0.18 [0.15 0.22], *p* <.001). Compared to individuals using smartphones running Android, iOS users were faster (β [CI] = −0.17 [−0.25 −0.09], *p* <.001). All effects except for the association between device type and DQRT were replicated in DQRTs estimated from Survey Set 2 (*N* = 991, Table S3). To test whether trails B game time (derived from Star Racer in Survey Set 1, *N* = 2,977) showed comparable associations to demographic variables as DQRT, we repeated the analyses with trails B game time as the independent variable. All effects were replicated in trails B except for the association between device type and trails B and the gender effect in individuals identifying as non-cisgender (Table S3). Using data from the EMA Set (*N* = 84), participants responded faster in the evening than in the morning (β [CI] = −0.56 [−0.69 −0.43], *p* <.001) across repeated assessments of EMA questions.

#### Association between DQRT and lifestyle, health, and mental health variables

**Fig. 4:**
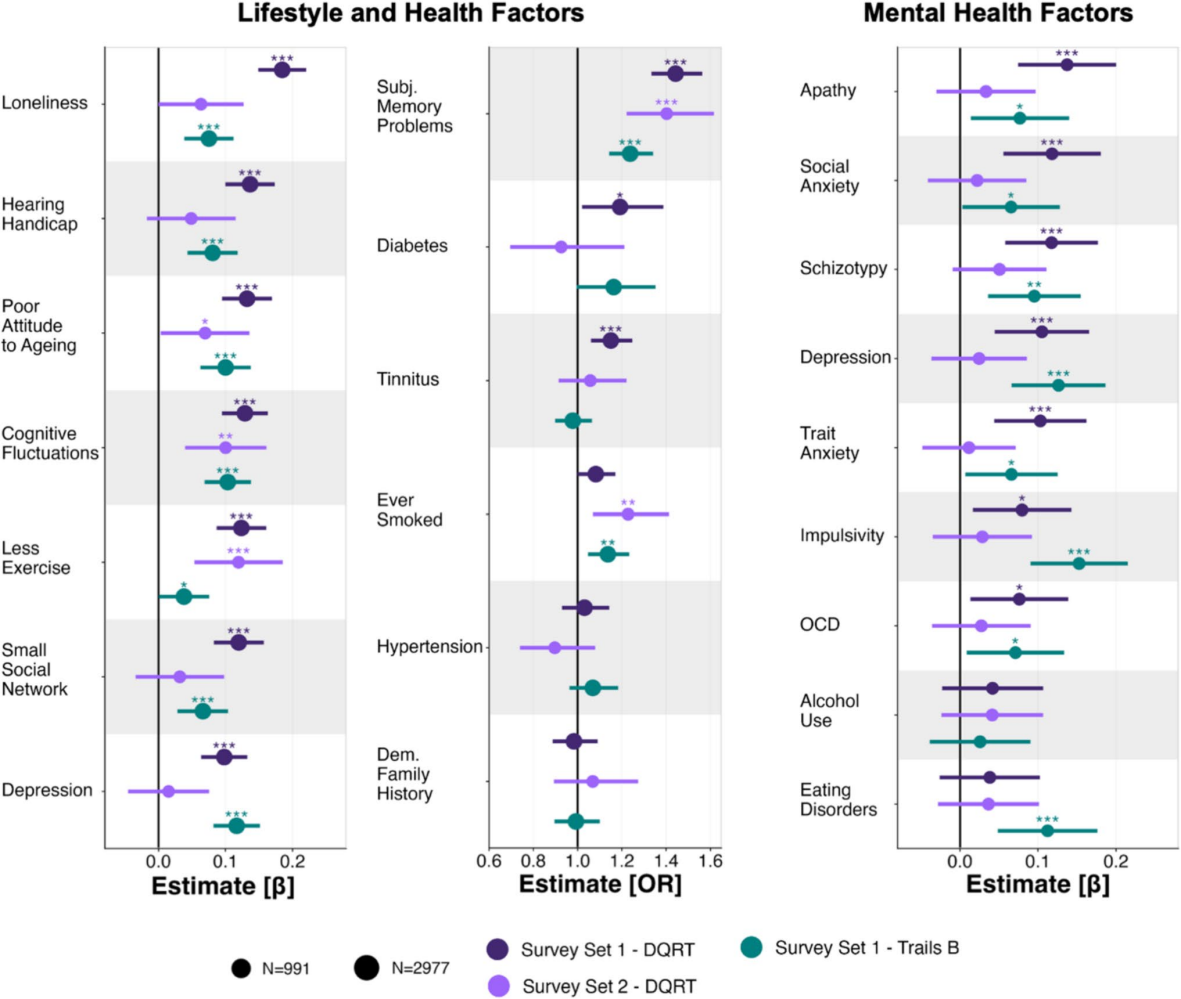
Digital questionnaire response time (DQRT) is linked to lifestyle, demographic, and health factors. Associations between DQRT (purple) and lifestyle, demographic, and health factors (left and middle columns) are comparable in magnitude or exceed the predictive performance of trails B game time (teal) when calculated from Survey Set 1 (*N* = 2,977, dark purple) but are less strong for DQRT calculated from Survey Set 2 (*N* = 991, light purple). DQRT was associated with mental health factors (derived from Survey Set 2, right column) when calculated from participants in Survey Set 1 who had also completed the mental health-related questionnaires (*N* = 991, dark purple) but not when calculated from Survey Set 2 (*N* = 991, light purple). Again, trails B game time (*N* = 991) showed an association with comparable or smaller magnitude except for impulsivity and eating disorders, where only trails B game time was predictive. To account for effects of age and gender, both variables were added as covariates. Point estimates are standardized beta coefficients for continuous dependent variables (left and right column) or odds ratios (OR) for binary dependent variables (middle column). Circle size indicates sample size for the respective analysis. Error bars depict 95% confidence intervals. * *p* <.05; ** *p* <.01; *** *p* <.001

To examine lifestyle, physical health, and mental health correlates of DQRT, we ran linear regression models (continuous independent variables; reporting standardized beta coefficients) and logistic regression models (binary independent variables; reporting odds ratios) predicting each individual difference measure separately (e.g., one model for loneliness, one for hearing handicap, and so on). In these models, mean DQRT and trails B game time derived from the largest dataset, Survey Set 1 (*N* = 2,977), were predictors of interest, and age and gender were covariates of no interest. For continuous outcomes, DQRT (0.10 ≤ β ≤ 0.18, all *p* <.001, Table S4, Fig. 4, left column, dark purple) and trails B (0.04 ≤ β ≤ 0.12, all *p* <.05, Table S4, Fig. 4, left column, teal) were significantly associated with all lifestyle and health factors. The associations were nominally stronger for DQRT than trails B in 6/7 cases. For binary lifestyle and health factors, DQRT was significantly associated with diabetes, tinnitus, and subjective memory problems (1.15 ≤ OR ≤ 1.44, all *p* <.05; Fig. 4, middle column, dark purple), but not smoking, whereas trails B game time was associated with subjective memory problems and smoking (1.14 ≤ OR ≤ 1.24, all *p* <.01; Fig. 4, middle column, teal), but not diabetes or tinnitus. Neither measure was associated with hypertension or dementia family history (Table S5). Turning to total scores on the mental health surveys in the subset who also completed them (*N* = 991), DQRT calculated from Survey Set 1 (Test) was associated with social anxiety, apathy, schizotypy, trait anxiety, depression, impulsivity, and OCD (0.08 ≤ β ≤ 0.14, all *p* <.05), but not alcohol use disorder or eating disorder symptoms (Table S4; Fig. 4, right column, dark purple). In comparison, trails B game time was linked to social anxiety, apathy, schizotypy, trait anxiety, impulsivity, eating disorders, depression, and OCD (0.07 ≤ β ≤ 0.15, all *p* <.05; Fig. 4, right column, teal), but not alcohol use disorder (Table S4).

These results concerned DQRTs estimated from Survey Set 1. We tested whether these effects held when calculating DQRTs based on Survey Set 2 (*N* = 991). Despite there being a gap between assessments and a smaller sample, we replicated the association with exercise, cognitive fluctuations, and attitude toward aging (0.07 ≤ β ≤ 0.12, all *p* <.05; Fig. 4, left column, light purple) and subjective memory problems (OR = 1.40, *p* <.001; Fig. 4, middle column, light purple). Associations with loneliness, hearing handicap, social network, and depression were not replicated (Table S4), nor were associations with diabetes or tinnitus (Table S5). A significant effect for smoking was observed, which was not present in the first dataset (Table S5). Turning to the mental health survey total scores, none of the seven original effects were replicated in this smaller sample (*N* = 991; all *p* >.05, Table S4).

#### The impact of questionnaire content on DQRT

The lack of replication of some individual difference variables across questionnaire sets could be due to sample size, but it also raises the issue of how strongly DQRT is affected by the content of the questionnaire used to generate it. To examine this, we first tested how the association between DQRT and cognitive test scores varied depending on the questionnaire used to estimate DQRT. We used linear models predicting trails B game time, model-based planning, and working memory, repeating the analysis 16 times using DQRTs calculated from a different questionnaire in each iteration. These questionnaires differed in both content and number of items (range 3–43); despite this, the results were remarkably consistent. DQRT showed a significant association with trails B game time in all 16 cases (Survey Set 1: 0.17 ≤ β ≤ 0.30; Survey Set 2: 0.29 ≤ β ≤ 0.36), even when estimated from just three items. The rank order of the association with the cognitive tests was preserved in all cases, with intermediate effect sizes for working memory (Survey Set 1: −0.006 ≤ β ≤ 0.13; Survey Set 2: 0.09 ≤ β ≤ 0.16) and smaller and mostly nonsignificant associations with model-based planning (Survey Set 1: −0.03 ≤ β ≤ 0.06; Survey Set 2: 0.02 ≤ β ≤ 0.08) (Fig. S8).

**Fig. 5:**
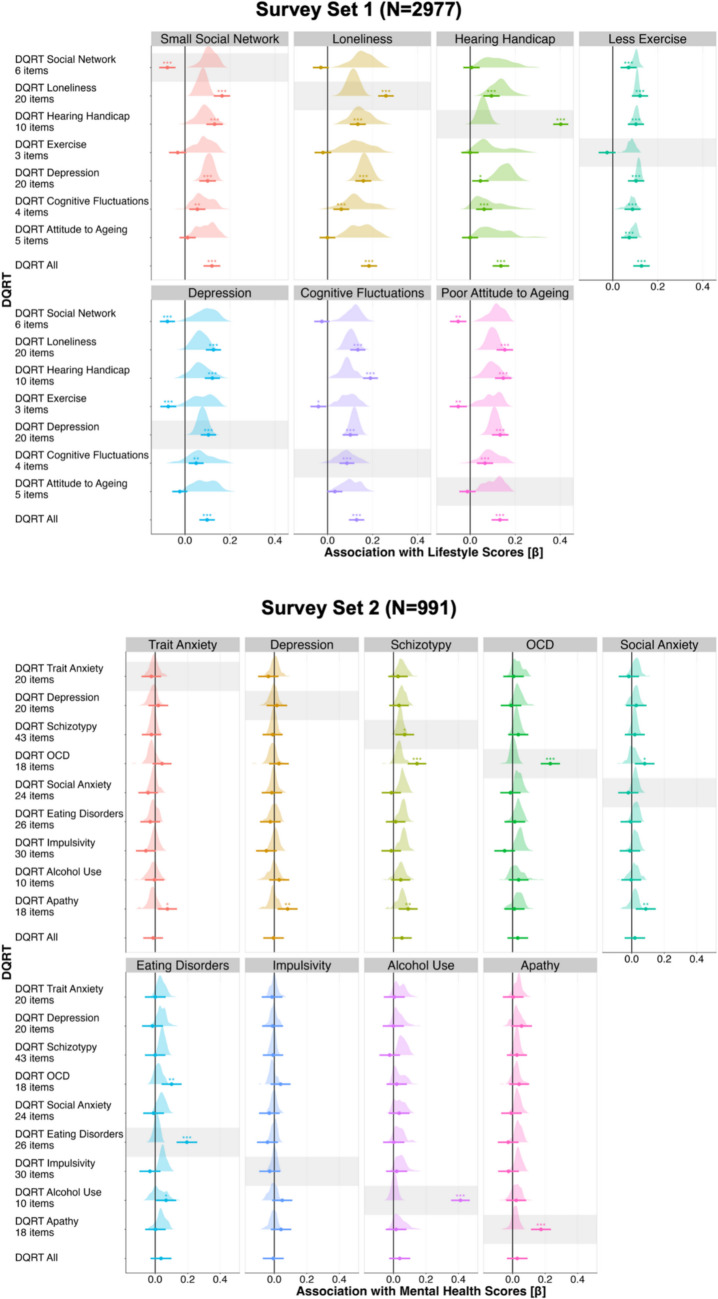
Digital questionnaire response time (DQRT) is biased by questionnaire content. Calculating the mean DQRT from each questionnaire separately results in higher predictive performance for the total scores of the respective questionnaire (gray-shaded area) for a subset of questionnaires. For the gray-shaded rows, point estimates (with 95% confidence intervals) that fall outside the distribution (reflecting the association between a questionnaire total score and DQRT calculated from the same number of items but redrawn 100 times from any other questionnaire in the respective part of the Neureka app) constitute evidence for bias. DQRTs calculated from Survey Set 1 (*N* = 2,977, top figure) showed an association with the respective scales’ total scores that exceeded the association with other total scores for the Lubben Social Network Scale (social network, red), UCLA (loneliness, yellow), HHIE-S (hearing handicap, green), and GODIN (less exercise, teal). DQRTs calculated from the AUDIT (alcohol use, purple), AES (apathy, pink), EAT (eating disorders, light blue), and OCI-R (OCD, green) showed an increased association with the respective scales’ total scores compared to total scores of other questionnaires in Survey Set 2 (*N* = 991, bottom figure). To account for differences in exposure to the lifestyle and health factors included in this analysis, we included age and gender as covariates in each model. Point estimates are standardized beta coefficients, and error bars depict 95% confidence intervals. Asterisks indicate the level of statistical significance for comparing the association between the questionnaire total score and DQRT calculated from the same questionnaire against zero. Colored distributions show the association between the respective questionnaire total score and DQRT calculated from the same number of items but (re)drawn 100 times from any other questionnaire in the respective part of the Neureka app.* *p* <.05; ** *p* <.01; *** *p* <.001

Next, we repeated this procedure, testing how the association between DQRT and self-report individual difference measures varied depending on the questionnaire used to generate the DQRT. This analysis is of critical importance, because circularities can arise if a participant takes systematically more time to complete a survey, depending on how high they score on that survey. To quantify this sort of bias, we compared the observed association between each questionnaire’s DQRT and its own total score to the distribution of associations generated for 100 bootstrapped DQRT estimates that were generated from random items drawn from the other questionnaires in the set. We propose that estimates (reflecting the association between a questionnaire total score and DQRT calculated from the same questionnaire) that fall outside the bootstrapped distribution (reflecting the association between a questionnaire total score and DQRT calculated from the same number of items but redrawn 100 times from any other questionnaire in the respective part of the Neureka app) constitute evidence for bias (see gray-shaded rows in Fig. 5). For Survey Set 1, there was evidence for bias in four out of seven cases (Fig. 5, upper panel). The bias was positive for hearing handicap and loneliness, wherein people who scored high on those scales took a particularly long time to answer those same surveys. The effect was reversed for the Lubben Social Network Scale and exercise scales, where people who engaged in less exercise and had smaller social networks completed these scales faster. For Survey Set 2, there was evidence of bias in four out of nine questionnaires (Fig. 5, lower panel). DQRTs calculated from questionnaires measuring alcohol use, apathy, eating disorders, and OCD all showed an increased association with the respective scales’ total scores compared to total scores of other questionnaires.

## Discussion

Digital questionnaire response time (DQRT) is an increasingly common form of research paradata that some have suggested can serve as a proxy for cognitive functioning (Hernandez et al., 2023; Höhne et al., 2017; Junghaenel et al., 2022). Here, we use a large smartphone-based dataset with cross-sectional and longitudinal data from questionnaires, experience sampling, and three cognitive tasks to provide a comprehensive validation of DQRT. Based on our observations, we include a set of recommendations for the use of DQRT in research (Box 1).

**Box 1: **Recommendations for the use of DQRT in research

**Table:** 

1. Remove raw response times that are two standard deviations above your sample’s median response time.
2. A minimum of 22 items leads to a stable association between DQRT and cognitive processing speed (based on trails B game time).
3. To reduce the risk of bias, calculate DQRT independently of any questionnaires you wish to relate to DQRT and calculate DQRT based on multiple questionnaires.
4. Remove items with conditional responses, that is items which are only shown to the participant depending on their response to a previous item, before calculating mean DQRT.
5. Control for gender, education, socio-economic status, device type – including input type (touchscreen versus keyboard and mouse) and screen size –, and time of day in cross-sectional and longitudinal studies.
6. DQRT has substantial practice effects that should be addressed by modeling assessment number in time-series analysis.

In terms of cognition, DQRT was most strongly associated with the reaction-time-based outcomes of the Trail Making Test trails A, which is commonly linked to processing speed, and trails B, which is commonly linked to cognitive flexibility or task switching (Bowie & Harvey, 2006). To a lesser extent, DQRT was related to working memory, but not model-based planning. These results are in line with previous studies (Hernandez et al., 2023; Junghaenel et al., 2022; Roque et al., 2020) and support a cognitive characterization of DQRT as a measure of cognitive processing speed. Importantly, psychological research tends to equate tasks with psychological constructs while, commonly, different tasks are linked to one psychological construct and different psychological constructs are linked to one task (Poldrack et al., 2011). With a certain supply of task outcomes available in one study, it is therefore often more helpful to characterize a new measure with regard to its association with established tasks. Accordingly, DQRT is expected to reflect well-known correlation patterns among cognitive tasks, which is supported by our finding that DQRT is linked to both the trails game times and working memory performance (range of observed correlations between trails game times and working memory, *r* = 0.36–0.46) but not model-based planning (range of observed correlations between trails game times and model-based planning, *r* = 0.11–0.16). To further investigate the usefulness of this new measure, we can relate the magnitude of the observed association between DQRT and established cognitive tasks to the variance shared between two different cognitive tasks that are supposed to measure a similar construct. The magnitude of associations we found between the trails outcomes (trails A and B) and DQRT show an overlap with reported effect sizes for the association between trails outcomes and the digit symbol substitution task, a different speed-based cognitive task capturing processing speed and set-shifting ability (Bettcher et al., 2011). Standardized associations with the digit symbol substitution task reported in the literature ranged from *r* = 0.29 to *r* = 0.68 for trails A and from *r* = 0.42 to *r* = 0.75 for trails B (Amodio et al., 2002; Crowe et al., 2007; Fellows et al., 2017; Joy et al., 2003; Sanchez-Cubillo et al., 2009), while the associations we observed between DQRT and trails outcomes ranged from *r* = 0.30 to *r* = 0.39, with the upper confidence interval reaching *r* = 0.44. Hence, DQRT captures variance linked to cognitive processing speed on a similar magnitude as traditional task-based measures, albeit on the lower end of the range of reported effect sizes. This could be due to the lower complexity inherent to survey measures compared to task-based measures, which is supported by the generally lower effect sizes reported for the association between the digit symbol substitution task and trails A, the trail variant characterized by lower complexity and, accordingly, lower mental load (Sanchez-Cubillo et al., 2009). Our findings were consistent across samples and questionnaire sets, including common Likert-scale multiple-choice items and brief EMA-style slider items. We found that 22 items was the minimum number required to estimate DQRT across two sets of surveys, suggesting that reliable DQRT can be rapidly acquired in routine data.

DQRT and trails B game time both had substantial practice effects, which are expected to increase in magnitude with increasing assessment frequency (Van der Elst et al., 2013). We suggest that researchers, where possible, take assessment history into account by modeling assessment number in linear mixed models to reduce the impact of this on within-person analyses (Van der Elst et al., 2013). Overall, we found good test–retest reliability for DQRT. We first characterized it based on the correlation of two assessments per participant, over different time lags, and found it to be good in all cases, with a slight reduction in reliability when the interval between measurements was longer. In participants with 15 or more repeated EMA assessments, we estimated reliability across time and within individuals, as measured by the ICC. Reliability for DQRT was on a moderate level and somewhat lower than for the gamified trail making test, but with overlapping confidence intervals. The association between trails B game time and DQRT was of similar magnitudes within individuals and between individuals.

Like task-based measures, DQRT was affected by time of day and device type. As the time of day selected for completing an assessment could be indicative of individual preferences and because devices are not randomly assigned, researchers using DQRT should control for both in their analyses. Similarly, analyses should control for gender, education, and socioeconomic status. DQRT was linked to a range of lifestyle and mental health variables, with similar effect sizes compared to trails B game time. DQRT calculated from Survey Set 1 outperformed trails B game time in two out of 22 cases, that is, for loneliness and less exercise, and showed similar magnitudes of associations for the remaining 20 variables, as indicated by the overlapping confidence intervals. Still, the effects were overall small in magnitude, and several of the associations with mental health measures in particular did not replicate when DQRT was calculated from a second set of questionnaires (Survey Set 2). Even though this was the case, confidence intervals still overlapped with the Survey Set 1 and trails B outcomes in all variables, indicating that there was substantial variability across all three measures (DQRT from Survey Set 1 and 2, and trails B game time) in all mental health-related variables. In comparison, confidence intervals were smaller for associations with lifestyle and health variables when using DQRT calculated from Survey Set 1 or trails B game time, but not DQRT calculated from Survey Set 2. This difference in confidence interval size for lifestyle and health variables could be due to differences in power, as the sample size for Survey Set 2 was smaller than the sample for Survey Set 1 and trails B game time. In sum, the similarity between DQRT and trails B game time in the direction and effect sizes across associations with lifestyle and mental health variables further supports DQRT as a valid measure of cognitive processing speed, with potentially similar utility in neuropsychological testing.

We observed evidence for bias in DQRT based on questionnaire content. Specifically, DQRTs for some questionnaires were longer or shorter depending on one’s final score on that questionnaire. Although these biases were evident, DQRTs derived from all questionnaires were nonetheless valid, insofar as they correlated with trails B game time. Researchers aiming to use DQRT for individual difference studies must consider such biases, and we recommend a full separation of questionnaires used for the derivation of DQRTs from those with content that one wishes to relate to those same DQRTs (Box 1).

Overall, we have highlighted various forms of systematic bias that affect DQRT that are important for future research to consider. It is important to note that many more additional and unmeasured factors may also contribute to DQRT, including language proficiency or reading level such as in dyslexia and motor acuity, as well as factors that affect attention and effort such as environmental factors (e.g., distractors), individual differences in attentional control such as in attention-deficit/hyperactivity disorder (ADHD), disorders affecting cognitive performance such as stroke or neurodegeneration, or conscientiousness (De Boeck & Jeon, 2019; Kyllonen & Zu, 2016; Yan & Tourangeau, 2007). However, many of the same sources of systematic bias apply to cognitive test scores (Borghans et al., 2008; Scharfen et al., 2018). We therefore conclude that, much like cognitive test scores, cross-sectional associations with DQRT are useful but should be interpreted with caution. In particular, DQRT may lend itself to within-person and longitudinal research aiming to understand how cognition fluctuates over time, and following interventions.

This study had some limitations. First, we were restricted to digital timestamps with second-level precision only. Digital timestamps are commonly recorded with millisecond precision, which might result in more accurate DQRT, particularly for brief item formats like EMA. This precision might also lead to the development of minimum thresholds for trial inclusion, which our data did not support but which could be important for identifying inattentive responders in paid research. Indeed, our proposals for maximum thresholds might be further improved by modeling expected response times at the level of each questionnaire (Schneider et al., 2023a). Second, we analyzed data from an online sample where participants used their privately owned devices and completed the modules on their own without supervision. Consequently, the data could be affected by a multitude of environmental influences and individual differences in how participants understood the tasks or interacted with their phones to place their inputs. For instance, differences in screen size were not collected for this work but have been shown to affect test scores from digital versions of cognitive tasks (Passell et al., 2021). We did not explicitly model these potential influences on response times, for example, using parameters of descriptive response time distributions (Matzke & Wagenmakers, 2009), as we attempted to keep our models simple to obtain a conservative estimate of the association between DQRT and cognition. However, we replicated our results across different survey sets and samples, and the satisfactory reliability across outcomes indicate that data quality was sufficient. Third, while we followed a gold-standard approach of dividing our data into training and test sets and tested the generalizability of our findings to different sets of questionnaires, our findings would benefit from external validation to ensure they are not restricted to the properties of smartphone data provided by citizen scientists. Similarly, we assess the type of variability in cognitive processes captured by DQRT using three different cognitive tasks and conclude that a characterization of “cognitive processing speed” fits best with our results as well as previous reports. Still, this characterization, also in terms of its clinical implications, needs to be validated using independent (clinical) samples and different cognitive tasks assessing processing speed or reaction time more generally.

To summarize, we found that DQRT, the time needed to respond to a questionnaire item, can be used as a valid and reliable index of cognitive processing speed that can be gathered at unprecedented scale, unobtrusively, and repeatedly, during a variety of real-world digital behaviors. We replicate previously observed associations between DQRT and task-based cognition as well as lifestyle and mental health factors. For the first time, we report biases in DQRT and provide insight-driven recommendations on how to mitigate the effect of these limitations when calculating DQRT to enhance the reproducibility and comparability of findings in this emerging field of digital assessment tools.

## Supplementary Information

Below is the link to the electronic supplementary material.

Supplementary file1 (DOCX 3769 KB)

## Data Availability

Data, code, and materials for this research are available at 10.17605/OSF.IO/HNCSG. To obtain access to the data, researchers are required to submit a Data Request Form which is linked in the OSF repository.
