# Supplementary material for: Digital questionnaire response time (DQRT): A ubiquitous and low-cost digital assay of cognitive processing speed
Source: Behav Res Methods. 2025 Jun 17;57(7):200. doi: 10.3758/s13428-025-02727-x (PMC12174177; doi:10.3758/s13428-025-02727-x)
Supplement: Supplementary file 1 — Supplementary file1 (DOCX 3769 KB) [file 13428_2025_2727_MOESM1_ESM.docx]

**Digital questionnaire response time (DQRT): a ubiquitous and low-cost digital assay of cognitive processing speed**

**Supplementary Information**

Vanessa Teckentrup^1*^, Anna M Rosická^1^, Kelly R Donegan^1^, Eoghan Gallagher^1^, Anna K Hanlon^1^, & Claire M Gillan^1,2^

^1^ School of Psychology and Trinity College Institute of Neuroscience, Trinity College Dublin, Dublin 2, Ireland

^2^ Global Brain Health Institute, Trinity College Dublin, Dublin 2, Ireland

**Corresponding author***

Dr Vanessa Teckentrup, [vanessa.teckentrup@tcd.ie](mailto:vanessa.teckentrup@tcd.ie)

Trinity College Dublin, College Green, Dublin 2, Ireland

**TABLE OF CONTENTS**

[METHODS 3](#_Toc197678600)

[**Figure S1: Flowchart showing the conditions for allocating participants to the survey sets.** 3](#_Toc197678601)

[**Star Racer (Trail Making Test) – Trails A and Trails B** 4](#_Toc197678602)

[**Cannon Blast (Two-Step Task) – Model-based planning** 6](#_Toc197678603)

[**Memory Match (Visual Short-Term Memory Binding Task) – Working memory** 8](#_Toc197678604)

[RESULTS 11](#_Toc197678605)

[**Figure S5: Associations between Digital Questionnaire Response Time (DQRT) and gamified tasks measuring cognition by type of assessment.** 11](#_Toc197678606)

[**Figure S6: Distributions of mean DQRT overall (last row in each plot for Survey Sets 1 and 2) and mean DQRT for each questionnaire (remaining rows) in the survey sets brought forward for analyses.** 12](#_Toc197678607)

[**Figure S7: Mean number of items needed for a stable association between trails B and DQRT across increasing sample size.** 13](#_Toc197678608)

[**Figure S8: Digital Questionnaire Response Time (DQRT) is invariant to questionnaire content in its association with cognition.** 14](#_Toc197678609)

[**Table S1: Association between minimally processed DQRT (excluding only responses >900 s) and cognitive tasks including trails A, trails B, working memory, and model-based planning.** 15](#_Toc197678610)

[**Table S2: Association between DQRT and cognitive tasks including trails A, trails B, working memory, model-based planning, and response times (RT) for the working memory and model-based planning tasks in held-out test sets.** 16](#_Toc197678611)

[**Table S3: Association between DQRT and demographics and device type in Survey Set 2 and association between trails B and demographics and device type in Survey Set 1.** 18](#_Toc197678612)

[**Table S4: Association between DQRT and lifestyle, health, and mental health factors for continuous independent variables.** 20](#_Toc197678613)

[**Table S5: Association between DQRT and lifestyle, health, and mental health factors for binary independent variables.** 23](#_Toc197678614)

[REFERENCES 25](#_Toc197678615)

# METHODS


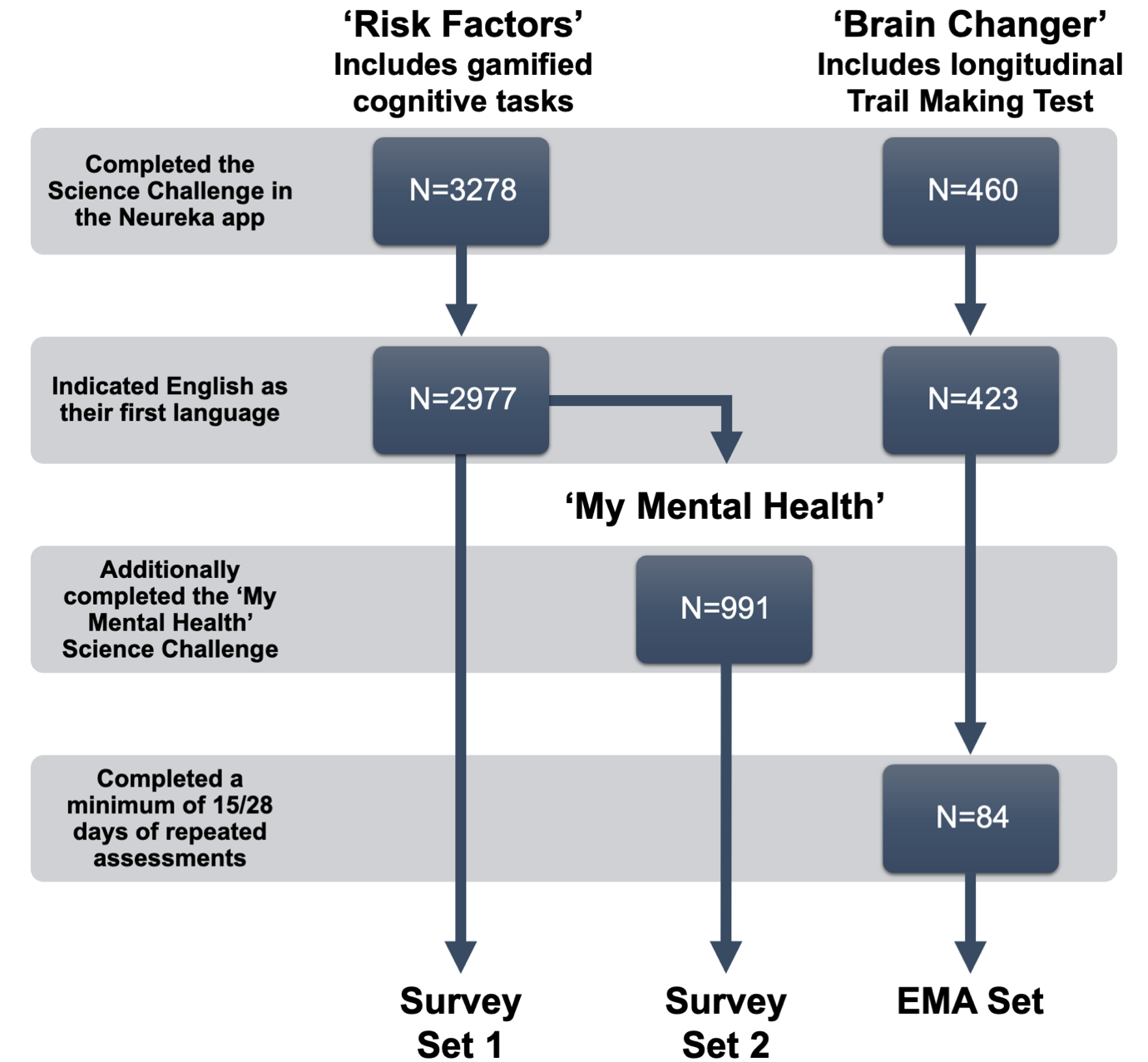


## **Figure S1: Flowchart showing the conditions for allocating participants to the survey sets.**

We included data from three science challenges, modules that contain surveys, quizzes, and gamified experimental tasks focusing on different aspects of brain health, in the Neureka research smartphone app. The ‘Risk Factors’ challenge included 12 questionnaires and three gamified cognitive tasks measuring cognitive processing speed, working memory and model-based planning. The ‘My Mental Health’ challenge included nine questionnaires, and the ’Brain Changer’ challenge included ecological momentary assessment twice a day in the morning and the evening as well as twice daily assessments of a gamified Trail Making Test (version B, measuring cognitive processing speed).

## **Star Racer (Trail Making Test) – Trails A and Trails B**

Star Racer is a gamified version of the Trail Making Test (TMT, Bowie & Harvey, 2006) that is commonly used to measure cognitive flexibility and processing speed as a facet of neuropsychological impairment in clinical settings.

A prior paper validated this task in detail (Rosická et al., 2023). In brief, performance on Star Racer trails A and trails B was correlated with completion time on the corresponding version A (*r* = 0.47, *p* = .002) or version B (*r* = 0.63, *p* < .001) of TMT. Internal consistency (Cronbach’s alpha) for mean completion time across runs was *α* [*CI*] = 0.81 [0.80, 0.83] for Star Racer trails A and *α* [*CI*] = 0.73 [0.71, 0.75] for Star Racer trails B, suggesting acceptable to good internal consistency. The test-retest reliability of mean completion time was good to moderate (*r* = 0.87, *p* < .001 for Star Racer trails A and *r* = .66, *p* < .001 for Star Racer trails B).


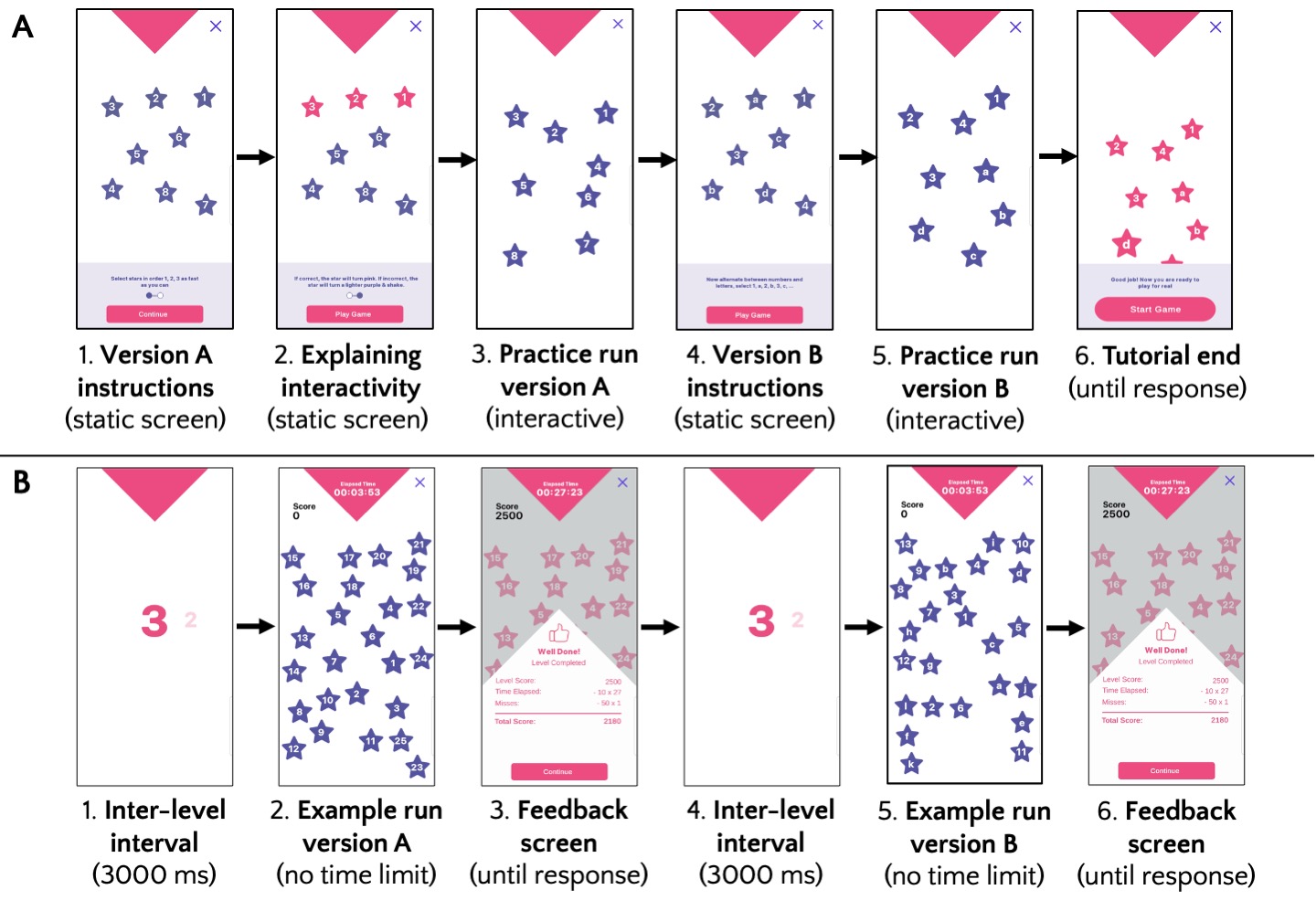


**Figure S2. Task structure of Star Racer, a gamified, smartphone-based version of the Trail Making Test (Spreen & Strauss, 1998) used to assess processing speed and cognitive flexibility.** While version A (select numbers in ascending order) of the task is commonly linked to processing speed and version B (alternate between numbers and letters in ascending order) has been proposed to reflect cognitive flexibility, the two versions are highly correlated, indicating an overlap in the cognitive processes they capture (Varjacic et al., 2018). A: Self-paced instruction screens and practice runs that appear at the start of Star Racer. B: Example of Star Racer runs (first A, then B version) with hard-coded star positions. Figure and legend reproduced with permission from Rosická et al. (2023) published in PsyArXiv preprints under the CC-BY-4.0 license (https://creativecommons.org/licenses/by/4.0/)

## **Cannon Blast (Two-Step Task) – Model-based planning**

Cannon Blast is a gamified version of the two-step reinforcement learning task (Daw et al., 2011) which estimates a participant’s ‘model-based’ tendency, representing the extent to which they make decisions using a mental map of action-outcome associations. In Cannon Blast, participants have 100 shots from a cannon to hit and collect as many diamonds as possible. To supply the cannon with balls to shoot, participants select from one of two containers with the left container having more purple-colored balls (80%) and the right container having more pink-colored balls (80%). Balls drawn from a container would either be good balls which could be used to collect the diamond (reward trial) or disintegrate upon firing, making it impossible to collect the diamond (unrewarded trial). However, even if the participant received a good ball, collecting the diamond was still dependent on their aim and timing. The probability that pink or purple balls are more likely to be good drifted over the course of the game and, thus, had to be constantly tracked by participants to increase their chance of collecting diamonds. In contrast to the original two-step task, Cannon Blast only presents participants with one stage of making a choice (which container to draw balls from). This was done to increase the importance of model-based contributions in this first-stage choice (Donegan et al., 2023).

Participants first completed a passive tutorial that explains the task and the goal to collect as many diamonds as possible. Then, participants completed 2 blocks of 100 trials each. The first block reflects an easy difficulty level with a static or slowly moving diamond, or static barriers that limited the participants aim. The second block reflects a medium difficulty level with a faster moving diamond and moving barriers. The outcome of interest for Cannon Blast is the model-based index (MBI) which is calculated using a hierarchical logistic regression model, predicting the binary choice between the two containers as a function of reward (good or bad ball) and transition (which color was shot from the chosen container). The model-based index reflects in how far participants use their knowledge about previous transitions and rewards to make a subsequent choice.

A prior paper validated this task in detail (Donegan et al., 2023). In brief, there was a moderate positive association between MBI derived from Cannon Blast and the traditional two-step reinforcement learning task (*r* = 0.40, *p* = .002). Split-half reliability for MBI were high for both the traditional task (*r* [*CI*] = 0.81 [0.70-0.88], *p* < .001) and Cannon Blast (*r* [*CI*] = 0.78 [0.66-0.87], *p* < .001). The test-retest reliability was *r* (423) = 0.63 assessed over a variable interval (median 4 days).

**Figure S3. Task structure of Cannon Blast, a smartphone game to assess model-based planning.** A. In this game, participants’ goal is to shoot as many diamonds as possible before their total number of shots (100 per block) runs out. To do so, they must aim a central cannon and then select which circular container to draw from. B. Purple and pink balls dynamically bounce around each of the flanked containers which depict the probability of a pink or purple ball being released. For example, the left container displays 8 purple balls and releases a purple ball 80% of the time (‘common’ transition) and displays 2 pink balls, giving a pink ball on 20% of trials (‘rare’ transition). C. The purple and pink balls have different values that dynamically change throughout the game. The value of the ball is defined as the probability of it being a ‘good ball’, i.e., one that remains intact after firing (rewarding trial), or a ‘dud ball’ (non-rewarding trial) that explodes shortly after being fired, and therefore cannot reach the diamond. D. We included 2 drifting reward probabilities (A, B) that quantitively differed on various metrics. Participants were randomly assigned a reward drift set at each block leading to four distinct drift set combinations (A-A, A-B, B-A, B-B). Figure and legend reproduced with permission from Donegan et al. (2023) published in Communications Psychology under the CC-BY-4.0 license (https://creativecommons.org/licenses/by/4.0/).

## **Memory Match (Visual Short-Term Memory Binding Task) – Working memory**

Visual working memory performance was assessed using ‘Memory Match’, a game loosely based on a previously published visual short-term memory binding task (VSMBT) (Parra et al., 2010). In Memory Match, participants are shown a set of symbols to memorize which they subsequently need to reidentify from a 4x5 grid. Participants either need to only remember the shape of the symbols (non-binding condition) or the color and the shape of the symbols (binding condition). Additionally, Memory Match uses two different stimulus types (letters and abstract shapes) and includes three levels of difficulty as participants need to memorize two (easy), three (medium), or four (hard) symbols at once. Participants collect points for selecting a correct shape from the grid and loose lives (with the number of lives equaling the number of target shapes for the trial) for making a mistake. A trial ends once a participant has selected all target shapes or lost all their lives.

Participants start with a passive tutorial with on-screen instructions, after which participants complete 24 trials consisting of two repeats of every trial type (two binding conditions x two stimulus types x three difficulty levels).

A prior paper validated this task in detail (Rosická et al., 2023). In brief, there was a moderate positive association between overall accuracy derived from Memory Match and the traditional VSMBT (*r* = 0.40, *p* = .023). Split-half reliability for overall accuracy were also moderate for both Memory Match (*r* = 0.64, *p* < .001; increasing to *r* = .78 after adjusting for test-length effects using the Spearman-Brown formula) and the traditional task (*r* = 0.60, *p* < .001; increasing to *r* = .75 after adjusting for test-length effects using the Spearman-Brown formula). The test-retest reliability for Memory Match was *r* = 0.63 (*p* < .001).


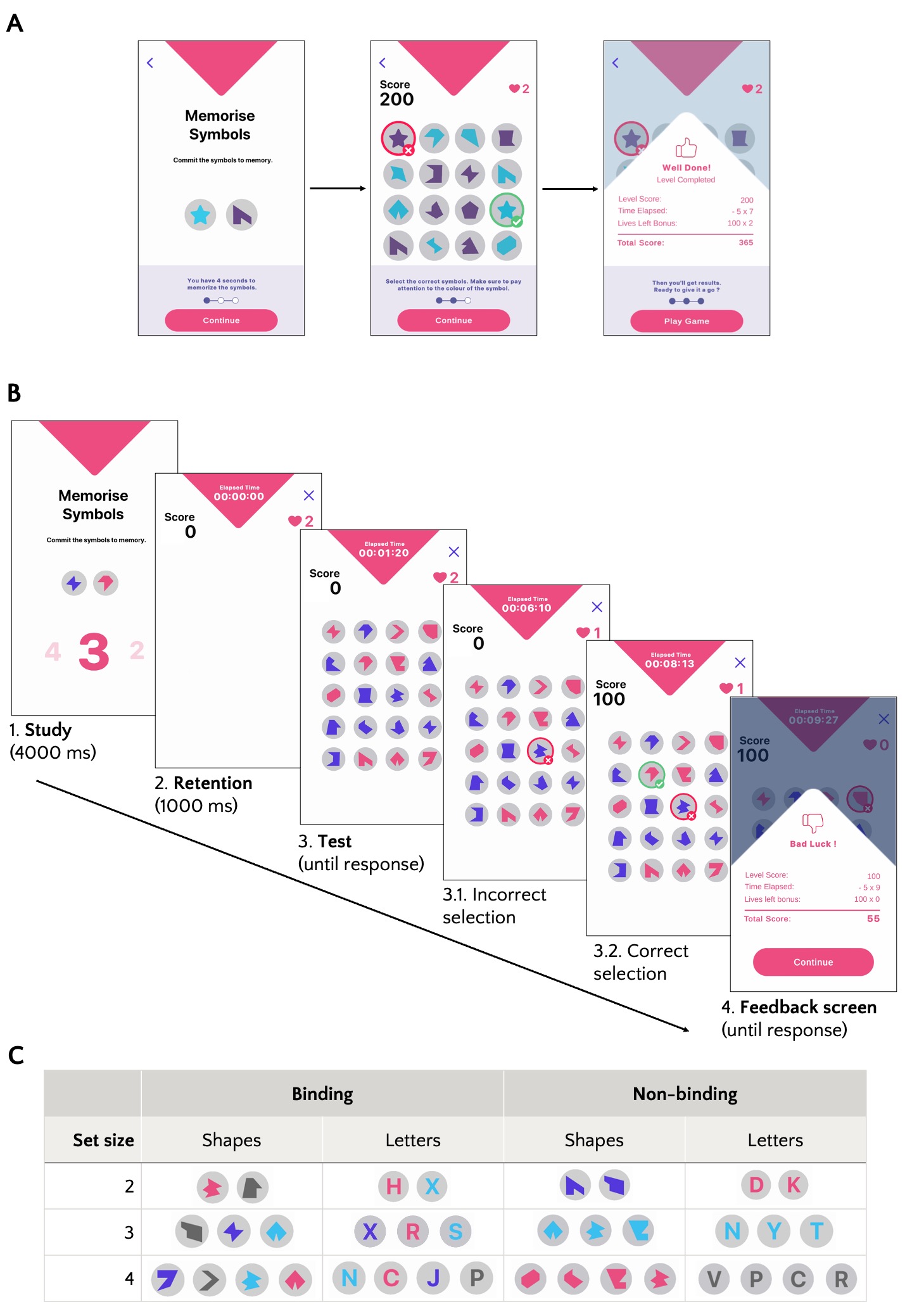


**Figure S4. Task structure of Memory Match, a smartphone game to assess visual working memory performance.** A: Self-paced task instruction screens that appear at the start of Memory Match. B: An example trial of Memory Match. The presentation of the study array (1.) is followed by a retention interval (2.) until the test display (3.) fully loads. Participants can lose lives and points by making incorrect selections (3.1.) or earn points by making correct selections (3.2.). Each trial is concluded by a feedback screen (4.). C: Examples of stimuli used in different trial types of Memory Match. Figure and legend reproduced with permission from Rosická et al. (2023) published in PsyArXiv preprints under the CC-BY-4.0 license (https://creativecommons.org/licenses/by/4.0/)

**RESULTS**


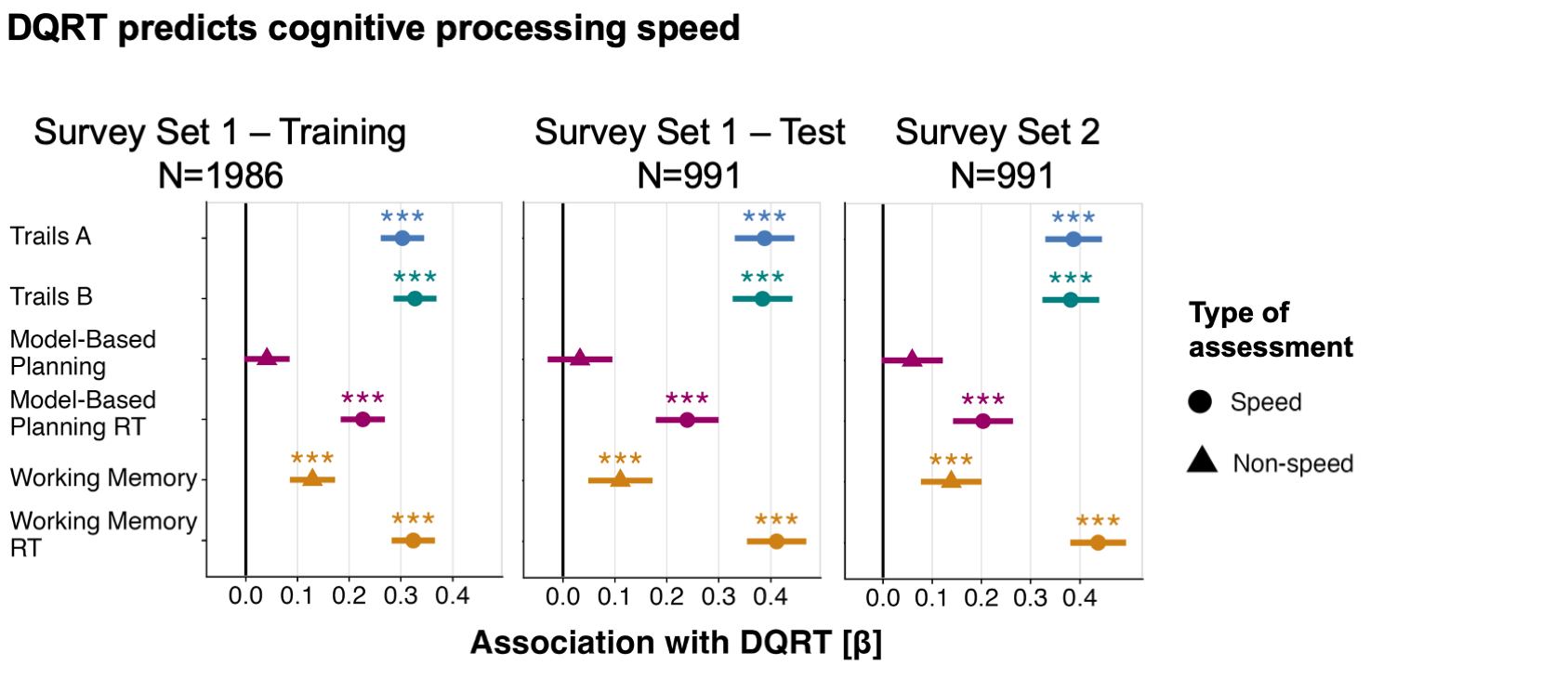


## **Figure S5: Associations between Digital Questionnaire Response Time (DQRT) and gamified tasks measuring cognition by type of assessment.**

Across the training set (‘Survey Set 1 – Training’), the held-out test set (‘Survey Set 1 – Test’) and when calculated from a separate set of questionnaires (‘Survey Set 2’), DQRT predicted the speed-based measures (circles) trails A, trails B, and the reaction times of participants on the tasks measuring working memory and model-based planning. For non-speed-based measures (triangles), DQRT predicted working memory (accuracy-based measure) but not model-based planning (computational estimate of a participant’s ‘model-based’ tendency). Point estimates are standardized beta coefficients and error bars depict 95% confidence intervals.

Annotation: * = p < .05; ** = p < .01; *** = p < .001


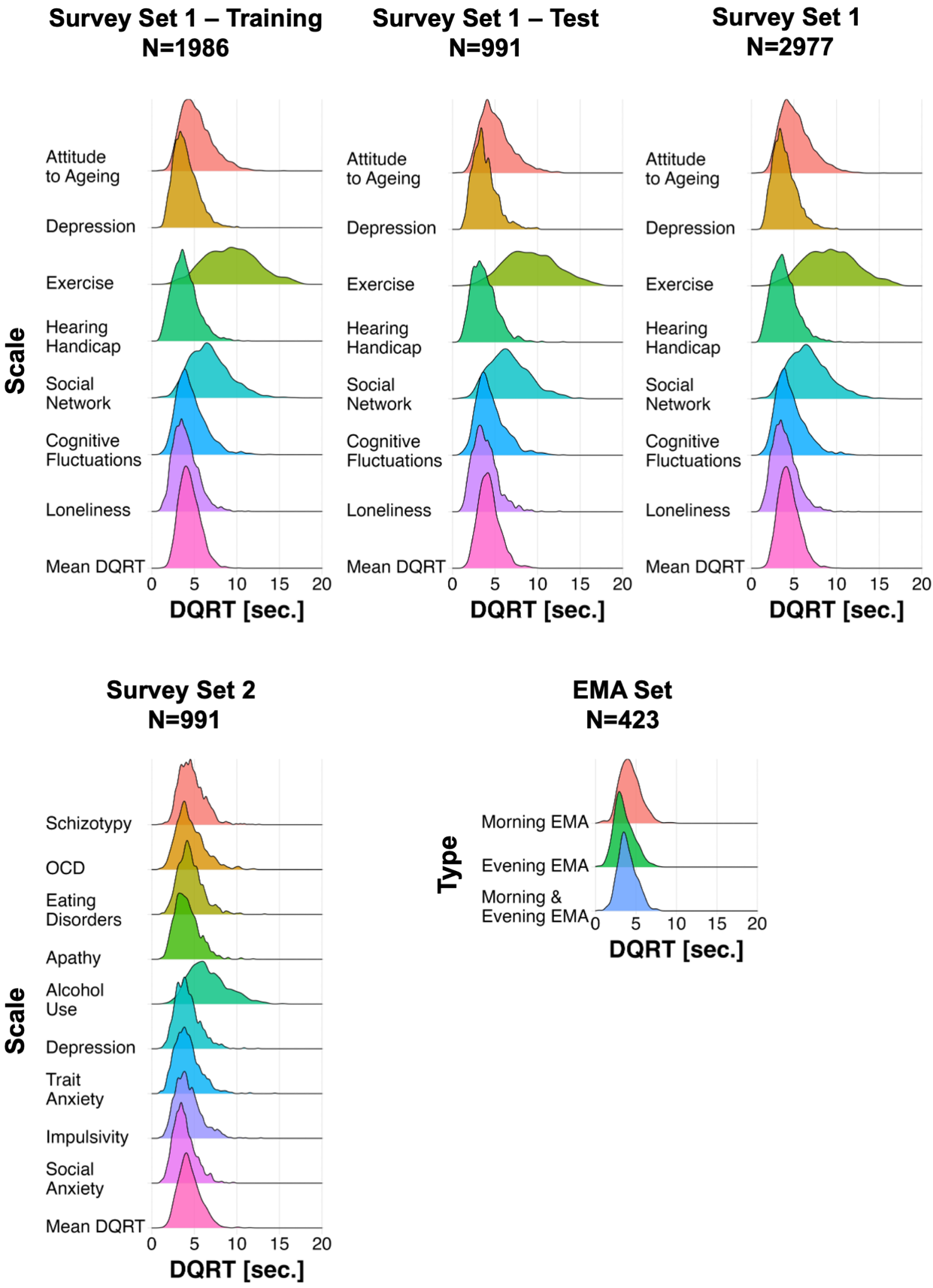


## **Figure S6: Distributions of mean DQRT overall (last row in each plot for Survey Sets 1 and 2) and mean DQRT for each questionnaire (remaining rows) in the survey sets brought forward for analyses.**

Density plots show the distribution of mean DQRT values across individuals in each survey set and associated sample. For Survey Set 1 and Survey Set 2, mean DQRT is shown for each scale that is part of the respective survey set separately while for the EMA Set, distributions of mean DQRT are presented separately for EMA items that were asked in the morning and EMA items that were asked in the evening compared to the combination of both.

**
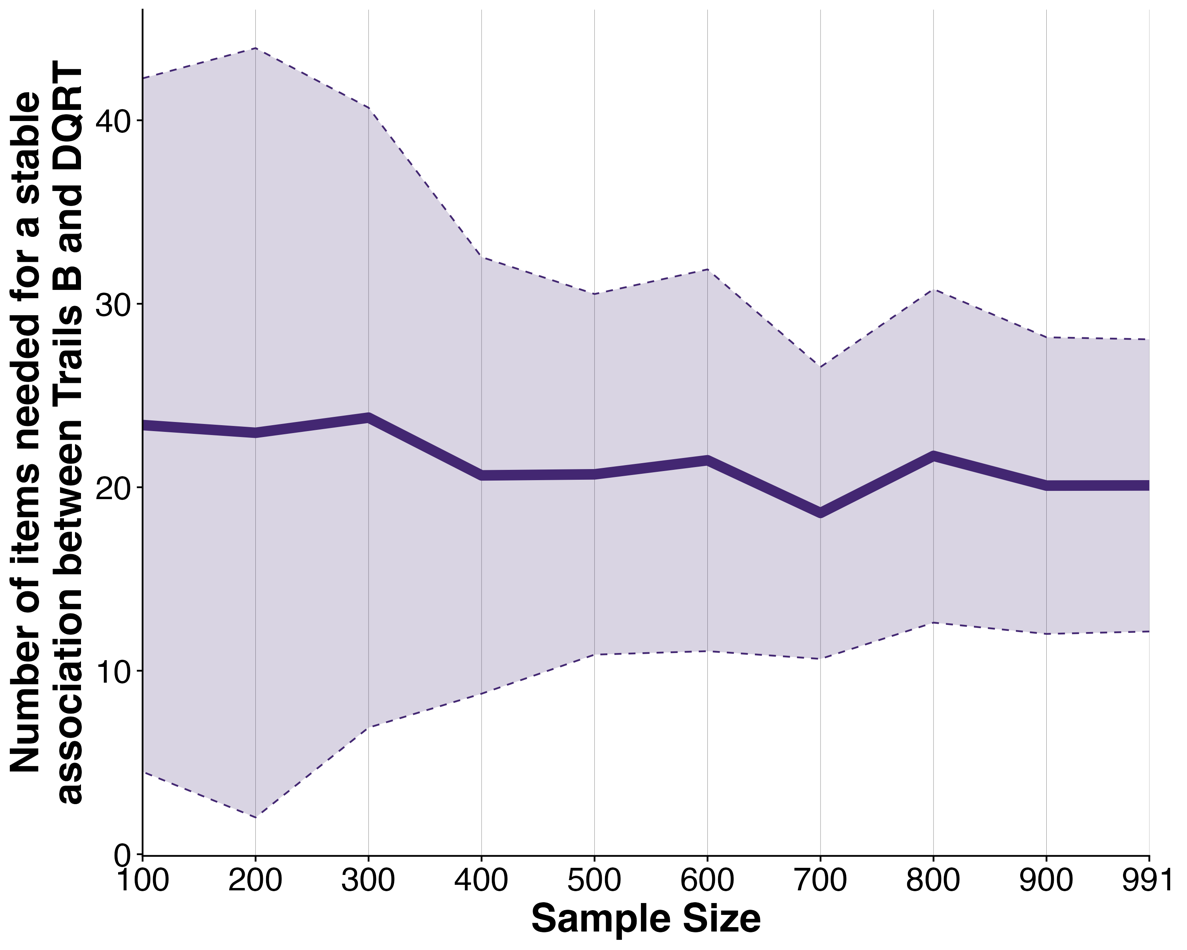
**

## **Figure S7: Mean number of items needed for a stable association between trails B and DQRT across increasing sample size.**

Samples ranging from N=100 to the full N=991 in steps of 100 were bootstrapped 100 times each from Survey Set 2. For each sample, we iteratively added survey items before calculating the association between trails B game time and DQRT. We then fitted an exponential function to calculate the number of iterative steps it takes for the curve reflecting the association between trails B game time and DQRT to reach 95% of its estimated asymptote which reflects the number of items needed to achieve a stable association. The purple line reflects the mean number of items needed across the 100 repetitions per sample size. The broken line depicts the standard deviation reflecting the uncertainty around the number of items needed.

**
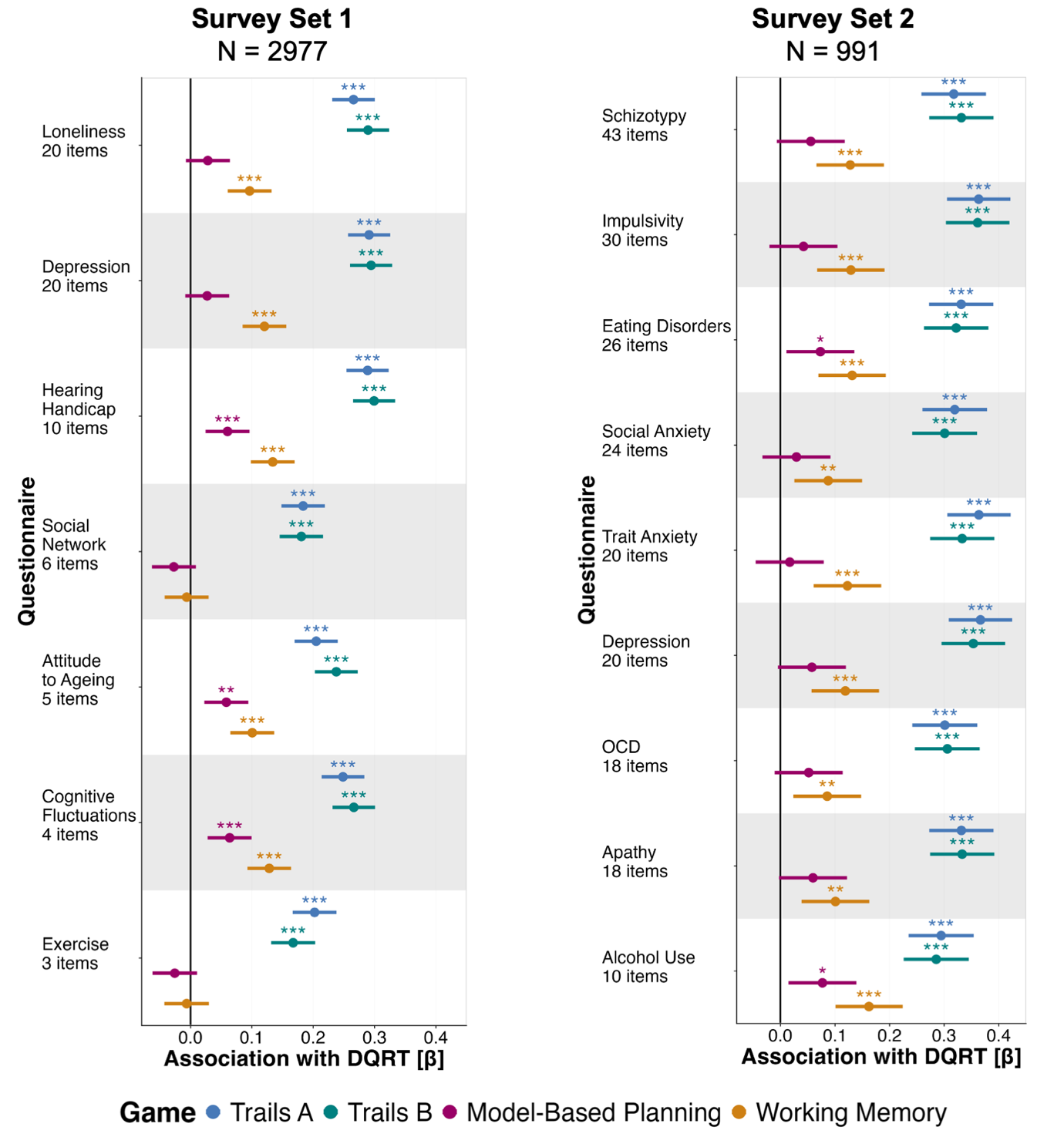
**

## **Figure S8: Digital Questionnaire Response Time (DQRT) is invariant to questionnaire content in its association with cognition.**

Associations between DQRT and cognition show the same rank order when mean DQRT is calculated separately for each questionnaire in Survey Set 1 (left column, *N*=2977) and Survey Set 2 (right column, *N*=991). Point estimates are standardized beta coefficients and error bars depict 95% confidence intervals.

Annotation: * = p < .05; ** = p < .01; *** = p < .001

## **Table S1: Association between minimally processed DQRT (excluding only responses >900 s) and cognitive tasks including trails A, trails B, working memory, and model-based planning.**

To assess further speed-based measures, response times (RT) for the working memory and model-based planning tasks are included as well.

| **Measure** | **Coefficient [*β*]** | **CI lower** | **CI upper** | ***p*** |
| --- | --- | --- | --- | --- |
| Trails A | 0.25 | 0.21 | 0.28 | <.001*** |
| Trails B | 0.25 | 0.21 | 0.28 | <.001*** |
| Working memory | 0.10 | 0.06 | 0.14 | <.001*** |
| Model-based planning | 0.007 | -0.03 | 0.04 | .71 |
| Working memory RT | 0.25 | 0.22 | 0.29 | <.001*** |
| Model-based planning RT | 0.23 | 0.19 | 0.26 | <.001*** |

Abbreviation: CI = 95% confidence interval

**Table S2: Association between DQRT and cognitive tasks including trails A, trails B, working memory, model-based planning, and response times (RT) for the working memory and model-based planning tasks in held-out test sets.**

‘Survey Set 1 – Test’ uses data from individuals (*N*=991) who completed surveys concerning risk factors for dementia in the Neureka app on the same occasion as they completed the cognitive tests. The same held-out sample (*N*=991) also completed items assessing mental health symptoms in a different section of the app (‘Survey Set 2’). A separate set of individuals (*N*=423) completed much shorter ecological momentary assessment (EMA; ‘EMA Set’) items in a third section of the app.

| **Measure** | **Coefficient [*β*]** | **CI lower** | **CI upper** | ***p*** |
| --- | --- | --- | --- | --- |
| **Survey Set 1 – Test (*N*=991, *N*_items_=68)** | | | | |
| Trails A | 0.39 | 0.33 | 0.45 | <.001*** |
| Trails B | 0.38 | 0.33 | 0.44 | <.001*** |
| Working memory | 0.11 | 0.05 | 0.17 | <.001*** |
| Model-based planning | 0.03 | -0.03 | 0.09 | .31 |
| Working memory RT | 0.41 | 0.35 | 0.47 | <.001*** |
| Model-based planning RT | 0.24 | 0.18 | 0.30 | <.001*** |
| **Survey Set 2 (*N*=991, *N*_items_=209)** | | | | |
| Trails A | 0.39 | 0.33 | 0.44 | <.001*** |
| Trails B | 0.38 | 0.32 | 0.44 | <.001*** |
| Working memory | 0.14 | 0.08 | 0.20 | <.001*** |
| Model-based planning | 0.06 | -0.004 | 0.12 | .07 |
| Working memory RT | 0.44 | 0.38 | 0.49 | <.001*** |
| Model-based planning RT | 0.20 | 0.14 | 0.26 | <.001*** |
| **EMA Set (*N*=423) – EMA items measured in the morning (*N*_items_=17)** | | | | |
| Trails B | 0.28 | 0.19 | 0.37 | <.001*** |
| **EMA Set (*N*=423) – EMA items measured in the evening (*N*_items_=14)** | | | | |
| Trails B | 0.23 | 0.13 | 0.32 | <.001*** |
| **EMA Set (*N*=423) – Mean across all EMA items (*N*_items_=31)** | | | | |
| Trails B | 0.29 | 0.20 | 0.38 | <.001*** |

Abbreviation: CI = 95% confidence interval

## **Table S3: Association between DQRT and demographics and device type in Survey Set 2 and association between trails B and demographics and device type in Survey Set 1.**

| **Measure** | **Coefficient [*β*]** | | **CI lower** | **CI upper** | ***p*** |
| --- | --- | --- | --- | --- | --- |
| **Survey Set 1 – DQRT (*N*=2977)** | | | | | |
| Gender (female - *male*) | -0.09 | -0.12 | | -0.05 | <.001*** |
| Gender (non-cisgender - *male*) | -0.13 | -0.26 | | -0.01 | .03* |
| Gender (prefer not to tell - *male*) | -0.02 | -0.40 | | 0.37 | .92 |
| Education | 0.17 | 0.13 | | 0.20 | <.001*** |
| Socio-economic status | 0.18 | 0.15 | | 0.22 | <.001*** |
| OS (iOS - *Android*) | -0.17 | -0.25 | | -0.09 | <.001*** |
| OS (other - *Android*) | 0.01 | -0.69 | | 0.72 | .97 |
| **Survey Set 1 – Trails B (*N*=2977)** | | | | | |
| Gender (female - *male*) | -0.04 | -0.08 | | -0.004 | .03* |
| Gender (non-cisgender - *male*) | -0.11 | -0.24 | | 0.008 | .07 |
| Gender (prefer not to tell - *male*) | -0.03 | -0.41 | | 0.35 | .86 |
| Education | 0.15 | 0.12 | | 0.18 | <.001*** |
| Socio-economic status | 0.09 | 0.06 | | 0.13 | <.001*** |
| OS (iOS - *Android*) | 0.06 | -0.02 | | 0.14 | .17 |
| OS (other - *Android*) | 0.11 | -0.60 | | 0.81 | .77 |
| **Survey Set 2 – DQRT (*N*=991)** | | | | | |
| Gender (female - *male*) | -0.08 | -0.15 | | -0.02 | .01* |
| Gender (non-cisgender - *male*) | -0.42 | -0.64 | | -0.20 | <.001*** |
| Gender (prefer not to tell - *male*) | -0.33 | -1.26 | | 0.59 | .48 |
| Education | 0.18 | 0.12 | | 0.24 | <.001*** |
| Socio-economic status | 0.14 | 0.08 | | 0.20 | <.001*** |
| OS (iOS - *Android*) | -0.01 | -0.15 | | 0.12 | .86 |
| OS (other - *Android*) | -0.32 | -2.18 | | 1.53 | .73 |

Abbreviation: CI = 95% confidence interval

## **Table S4: Association between DQRT and lifestyle, health, and mental health factors for continuous independent variables.**

Results for linear regression models (continuous independent variables; reporting standardized beta coefficients) predicting total scores of surveys assessing lifestyle, health, and mental health factors with mean DQRT (Survey Set 1 – Training (*N*=2977); Survey Set 1 – Test (*N*=991); Survey Set 2 (*N*=991)) and trails B (*N*=2977 for Survey Set 1 and *N*=991 for Survey Set 2) as predictors of interest, and age and gender as covariates of no interest.

| **Measure** | **Coefficient [*β*]** | **CI lower** | **CI upper** | ***p*** |
| --- | --- | --- | --- | --- |
| **Survey Set 1 (Training) – DQRT (*N*=2977, *N*_items_=68)** | | | | |
| Loneliness | 0.18 | 0.15 | 0.22 | <.001*** |
| Hearing handicap | 0.14 | 0.10 | 0.17 | <.001*** |
| Poor attitude to aging | 0.13 | 0.09 | 0.17 | <.001*** |
| Cognitive fluctuations | 0.13 | 0.09 | 0.16 | <.001*** |
| Less exercise | 0.12 | 0.09 | 0.16 | <.001*** |
| Small social network | 0.12 | 0.08 | 0.16 | <.001*** |
| Depression (CES-D) | 0.10 | 0.06 | 0.13 | <.001*** |
| **Survey Set 1 (Test) – DQRT (*N*=991, *N*_items_=209)** | | | | |
| Apathy | 0.14 | 0.07 | 0.20 | < .001*** |
| Alcohol Use | 0.04 | -0.02 | 0.11 | .206 |
| Impulsivity | 0.08 | 0.02 | 0.14 | .013* |
| Eating Disorders | 0.04 | -0.03 | 0.10 | .244 |
| Social Anxiety | 0.12 | 0.06 | 0.18 | < .001*** |
| OCD | 0.08 | 0.01 | 0.14 | .017* |
| Schizotypy | 0.12 | 0.06 | 0.18 | < .001*** |
| Depression | 0.11 | 0.04 | 0.17 | .001*** |
| Trait Anxiety | 0.10 | 0.04 | 0.16 | .001*** |
| **Survey Set 1 – Trails B (*N*=2977, *N*_items_=68)** | | | | |
| Loneliness | 0.08 | 0.04 | 0.11 | <.001*** |
| Hearing handicap | 0.08 | 0.04 | 0.12 | <.001*** |
| Poor attitude to aging | 0.10 | 0.06 | 0.14 | <.001*** |
| Cognitive fluctuations | 0.10 | 0.07 | 0.14 | <.001*** |
| Less exercise | 0.04 | 0.0004 | 0.08 | .048* |
| Small social network | 0.07 | 0.03 | 0.10 | <.001*** |
| Depression (CES-D) | 0.12 | 0.08 | 0.15 | <.001*** |
| **Survey Set 1 – Trails B (*N*=991, *N*_items_=209)** | | | | |
| Apathy | 0.08 | 0.01 | 0.14 | .017* |
| Alcohol Use | 0.03 | -0.04 | 0.09 | .432 |
| Impulsivity | 0.15 | 0.09 | 0.22 | < .001*** |
| Eating Disorders | 0.11 | 0.05 | 0.18 | .001*** |
| Social Anxiety | 0.07 | 0.00 | 0.13 | .040* |
| OCD | 0.07 | 0.01 | 0.13 | .026* |
| Schizotypy | 0.10 | 0.04 | 0.15 | .002** |
| Depression | 0.13 | 0.07 | 0.19 | < .001*** |
| Trait Anxiety | 0.07 | 0.01 | 0.13 | .029* |
| **Survey Set 2 – DQRT (*N*=991, *N*_items_=209)** | | | | |
| Loneliness | 0.06 | -0.00008 | 0.13 | .05 |
| Hearing handicap | 0.05 | -0.02 | 0.12 | .14 |
| Poor attitude to aging | 0.07 | 0.003 | 0.14 | .04* |
| Cognitive fluctuations | 0.10 | 0.04 | 0.16 | .001** |
| Less exercise | 0.12 | 0.05 | 0.19 | <.001*** |
| Small social network | 0.03 | -0.03 | 0.10 | .34 |
| Depression (CES-D) | 0.02 | -0.05 | 0.08 | .62 |
| Apathy | 0.03 | -0.03 | 0.10 | .30 |
| Alcohol Use | 0.04 | -0.02 | 0.11 | .21 |
| Impulsivity | 0.03 | -0.03 | 0.09 | .38 |
| Eating Disorders | 0.04 | -0.03 | 0.10 | .27 |
| Social Anxiety | 0.02 | -0.04 | 0.09 | .49 |
| OCD | 0.03 | -0.04 | 0.09 | .39 |
| Schizotypy | 0.05 | -0.01 | 0.11 | .10 |
| Depression | 0.02 | -0.04 | 0.09 | .43 |
| Trait Anxiety | 0.01 | -0.05 | 0.07 | .70 |

Abbreviation: CI = 95% confidence interval

## **Table S5: Association between DQRT and lifestyle, health, and mental health factors for binary independent variables.**

Results for logistic regression models (binary independent variables; reporting odds ratios (OR)) predicting total scores of surveys assessing lifestyle, health, and mental health factors with mean DQRT (Survey Set 1 (*N*=2977); Survey Set 2 (*N*=991)) and trails B (Survey Set 1 (*N*=2977)) as predictors of interest, and age and gender as covariates of no interest.

| **Measure** | **Coefficient [*OR*]** | **CI lower** | **CI upper** | ***p*** |
| --- | --- | --- | --- | --- |
| **Survey Set 1 – DQRT (*N*=2977, *N*_items_=68)** | | | | |
| Subjective memory problems | 1.44 | 1.33 | 1.56 | <.001*** |
| Diabetes | 1.19 | 1.02 | 1.39 | 0.03* |
| Tinnitus | 1.15 | 1.06 | 1.25 | <.001*** |
| Ever smoked | 1.08 | 1.0 | 1.17 | .05 |
| Hypertension | 1.03 | 0.93 | 1.14 | .56 |
| Dementia family history | 0.98 | 0.89 | 1.09 | .75 |
| **Survey Set 1 – Trails B (*N*=2977)** | | | | |
| Subjective memory problems | 1.24 | 1.14 | 1.34 | <.001*** |
| Diabetes | 1.16 | 0.99 | 1.35 | .05 |
| Tinnitus | 0.98 | 0.90 | 1.06 | .61 |
| Ever smoked | 1.14 | 1.05 | 1.23 | .002** |
| Hypertension | 1.07 | 0.96 | 1.18 | .21 |
| Dementia family history | 0.99 | 0.89 | 1.10 | .89 |
| **Survey Set 2 – DQRT (*N*=991, *N*_items_=209)** | | | | |
| Subjective memory problems | 1.40 | 1.22 | 1.62 | <.001*** |
| Diabetes | 0.93 | 0.70 | 1.21 | .59 |
| Tinnitus | 1.06 | 0.91 | 1.22 | .45 |
| Ever smoked | 1.23 | 1.07 | 1.41 | .004** |
| Hypertension | 0.90 | 0.74 | 1.08 | .26 |
| Dementia family history | 1.07 | 0.89 | 1.27 | .47 |

Abbreviation: CI = 95% confidence interval

# REFERENCES

Bowie, C. R., & Harvey, P. D. (2006). Administration and interpretation of the Trail Making Test. *Nat Protoc*, *1*(5), 2277-2281. <https://doi.org/10.1038/nprot.2006.390>

Daw, N. D., Gershman, S. J., Seymour, B., Dayan, P., & Dolan, R. J. (2011). Model-based influences on humans' choices and striatal prediction errors. *Neuron*, *69*(6), 1204-1215. <https://doi.org/10.1016/j.neuron.2011.02.027>

Donegan, K. R., Brown, V. M., Price, R., Pringle, A., Gallagher, E., Hanlon, A., & Gillan, C. (2023). Using smartphones to optimise and scale-up the assessment of model-based planning. *PsyArXiv*. <https://doi.org/10.31234/osf.io/hpm4s>

Parra, M. A., Abrahams, S., Logie, R. H., Mendez, L. G., Lopera, F., & Della Sala, S. (2010). Visual short-term memory binding deficits in familial Alzheimer's disease. *Brain*, *133*(9), 2702-2713. <https://doi.org/10.1093/brain/awq148>

Rosická, A. M., Teckentrup, V., Fittipaldi, S., Ibanez, A., Pringle, A., Gallagher, E., Hanlon, A., Claus, N., McCrory, C., Lawlor, B., Naci, L., & Gillan, C. (2023). Modifiable dementia risk factors associated with objective and subjective cognition. *PsyArXiv*. <https://doi.org/10.31234/osf.io/rc4sy>

Spreen, O., & Strauss, E. (1998). *A compendium of neuropsychological tests : administration, norms, and commentary* (2nd ed.). Oxford University Press.

Varjacic, A., Mantini, D., Demeyere, N., & Gillebert, C. R. (2018). Neural signatures of Trail Making Test performance: Evidence from lesion-mapping and neuroimaging studies. *Neuropsychologia*, *115*, 78-87. <https://doi.org/10.1016/j.neuropsychologia.2018.03.031>
